# Growth Modeling of the Maternal Cytokine Milieu throughout Normal Pregnancy: Macrophage-Derived Chemokine Decreases as Inflammation/Counterregulation Increases

**DOI:** 10.1155/2015/952571

**Published:** 2015-03-17

**Authors:** Shernan G. Holtan, Yiyi Chen, Rajani Kaimal, Douglas J. Creedon, Elizabeth Ann L. Enninga, Wendy K. Nevala, Svetomir N. Markovic

**Affiliations:** ^1^Division of Hematology, Oncology, and Transplantation, University of Minnesota, 420 Delaware Street, MMC 480, Minneapolis, MN 55455, USA; ^2^Department of Public Health and Preventive Medicine, Oregon Health & Science University, 3181 SW Sam Jackson Park Road, Portland, OR 97239, USA; ^3^Department of Obstetrics and Gynecology, Mayo Clinic Graduate School of Medicine, 200 First Street SW, Rochester, MN 55905, USA; ^4^Department of Immunology, Mayo Clinic Graduate School of Medicine, 200 First Street SW, Rochester, MN 55905, USA; ^5^Division of Hematology, Department of Medicine, Mayo Clinic Graduate School of Medicine, 200 First Street SW, Rochester, MN 55905, USA; ^6^Department of Oncology, Mayo Clinic Graduate School of Medicine, 200 First Street SW, Rochester, MN 55905, USA

## Abstract

Several recent studies have shown differences in the maternal immune milieu at different phases of pregnancy, but most studies have been cross-sectional or of relatively few time points. Levels of 42 cytokines were determined using a multiplex bead-based assay on archived serum from a cohort of pregnant women (*N* = 16) at median of 18 time points tested, from the first trimester through to parturition, per woman. Unconditional growth modeling was then used to determine time-dependent changes in levels of these cytokines. Macrophage-derived chemokine (MDC, aka CCL22) decreases as pregnancy progresses. IL-1*β*, IL-6, IL-8, IL-12p70, IL-13, IL-15, IP-10, and FLT3-ligand increase as a function of gestational weeks, and IFN*α*2, IL-1ra, IL-3, IL-9, IL-12p40, and soluble CD40 ligand increase as a function of trimester. As pregnancy normally progresses, a maternal shift away from a type 2-biased immune response and toward an inflammatory/counterregulatory response is observed.

## 1. Introduction

The female immune system faces a delicate balancing act during pregnancy. On one hand, it must develop tolerance to paternal antigens to avoid a lethal immunologic attack against the fetus. On the other hand, it must preserve the ability to fight infections from a multitude of commensal and environmental pathogens. Disruption of this balance can have devastating consequences, including preterm labor and death of the fetus and/or mother. The normal maternal immune response to pregnancy is increasingly recognized as a dynamic process, with changes in the maternal pro/anti-inflammatory profile occurring at different stages of gestation [1–3]. Despite this recognition, a complete, longitudinal immunologic profile of normal pregnancy has not been performed. Such analyses are critical for understanding the response to specific infectious and immunologic diseases that show disproportionately negative outcomes during pregnancy, such as influenza [4] and ulcerative colitis [5].

One approach to such immune profiling is through the use of multiplex cytokine arrays, allowing for simultaneous quantification of many proteins with a very small amount of plasma or serum. Others have used multiplex arrays to study the maternal cytokine milieu, but the studies have been predominantly limited time point cross-sectional [6–8] or case-control [9–11] in design. The results of some longitudinal studies have been published in recent years, although results have been based on relatively few time points (5 or fewer) [1–3]. In this study, we applied the multiplex array approach to evaluate the changes in 42 cytokines in rich detail during pregnancy. The samples are from a cohort of 16 pregnant women, with each subject sampled a median of 18 times. Because this represents a significant increase in the number of tests per subject compared to previous studies, we also describe a strategy addressing within-subject correlation with repeated measurements using unconditional growth modeling. With this approach, we have been able to detail the typical longitudinal variation of serum cytokines throughout pregnancy in this cohort of women.

## 2. Materials and Methods

### 2.1. Participants

Archived longitudinal serum samples from 16 primigravid women were tested in this study. Each participant had a serum sample collected and cryopreserved biweekly from study enrollment during the first trimester (average of 9.7 weeks' gestation for first samples) until 34 weeks' gestation, then weekly from 34 weeks until delivery. As a result of this repeated sampling, a median of 18 samples per participant were obtained longitudinally throughout pregnancy. A total of 312 longitudinal pregnancy samples were tested. Samples were collected under a protocol approved by the Mayo Clinic Institutional Review Board (IRB) that accrued participants from 1987 to 1988. IRB approval was obtained for analysis of these archived samples and clinical data. Information regarding date of the blood draw, date of delivery, outcome of the delivery, and any major complications that the woman experienced during pregnancy (hypertensive disorders, preterm labor, and infections) was available and helped identify appropriate participants to include. Only women who carried their pregnancies to term and experienced no pregnancy complications were included in this study. We only used samples from subjects who had specimens obtained throughout the entire course of pregnancy and whose complete, longitudinally collected specimens appeared intact and nondesiccated. Most cytokines are stable in storage at −80 degrees C for up to 2 years [12], but stability beyond that time is not well known. Therefore, before proceeding with studies on the samples of interest, we first tested protein levels of a small number of individually stored serum aliquots from other subjects archived on this protocol. Since we found detectable levels of cytokines in the pregnant serum samples that were within range of control samples, we proceeded with the entire study involving a cohort of 16 healthy, primigravid women who completed a term pregnancy without any significant complications. Samples were assayed when freshly thawed to avoid freeze-thaw cycles, which are known to degrade cytokines [12].

Contemporary plasma samples from 11 nonpregnant control subjects were collected under a separate IRB-approved protocol for collection of biospecimens from healthy individuals within the same health system in 2010 and similarly assessed on the same 96-well plates for comparison and as a quality control measure. For contemporary nonpregnant control samples, peripheral venous blood was drawn into heparinized Vacutainer tubes that were processed and separated into plasma and peripheral blood mononuclear cells (PBMCs) following gradient centrifugation using Ficoll-Paque (GE Healthcare, Uppsala, Sweden). Plasma was collected and immediately frozen at −80°C in 1 mL aliquots until use.

### 2.2. Multiplex Serum and Plasma Cytokine Concentration Determination

Protein levels for 42 cytokines, chemokines, and growth factors (Table 1) were measured using the MILLIPLEX MAP Human Cytokine/Chemokine Kit (Millipore, Billerica, MA, USA) per manufacturer's instructions. To minimize the potential for interassay variability, each subject had all of their longitudinal specimens analyzed on the same plate. Plasma/serum samples were not diluted prior to incubation with fluorescently dyed microspheres. Protein concentrations were determined using a linear regression standard curve from each plate generated using the high PMT concentrations with sensitivity from 3.2 to 2,000 pg/mL. All samples were tested in duplicate with the mean value of the measurements used for statistical analyses.

### 2.3. Statistical Analysis

Comparison of baseline (first trimester) and end of pregnancy levels of cytokines/growth factors was carried out between the 16 primigravid subjects and the 11 nonpregnant control subjects using the Kruskal-Wallis tests. There were extremes of values that fell outside of the limits of the multiplex array. For cytokines below the limit of detection (LD), we assigned a value that was half the lowest detectable value for the assay as previously described [13]. Analytes above the detectable range were assigned a threshold concentration [14]. To obtain a visual representation of overall patterns in the highly dimensional raw data, we performed unsupervised hierarchical clustering according to Ward's method [15]. As an additional multivariate technique to identify patterns within the data, principle components analysis (PCA) was also completed on the raw values obtained from the multiplex array.

### 2.4. Data Normalization

To assess the within-subject correlation, we determined change of cytokines and growth factors from a baseline time point—the values obtained from the participants' first blood draw in the first trimester—by taking the log of 1 plus fold change from the baseline value: log⁡(1 + *x*), where *x* = (cytokine concentration of the sample of interest/cytokine concentration of the baseline sample) to analyze trends over time.

### 2.5. Longitudinal Analyses

First, we graphically assessed the growth trajectory for the 42 cytokines using smoothing splines. These plots were used to help visually determine within-subject variation over time. We then fit unconditional mean models (UMM) for the 42 cytokines in order to estimate the variance components: the within-subject variance (*σ*_*ε*_^2^) and the between-subject variance (*σ*_0_^2^). Estimating the two variance components helps to determine whether there is sufficient variation to warrant further analysis and enabled computations of the intraclass correlation coefficient, *ρ*, which describes the proportion of the total outcome variation that lies between subjects.

Next, we fit unconditional growth models (UGM), which allowed random slope but not random intercept because the starting value of all patients was the same (baseline value = 0.3) due to normalization to each individual's baseline value. As both gestational age in weeks and trimester can be used as the time covariate for growth curve models, and as the trimester can be treated as either a continuous or a categorical variable (depending on whether we assume linear relationship between trimester and outcome variables), six different models were fitted for each cytokines as follows: (1) model with only the gestational age in weeks as the time covariate, (2) model with only the trimester (continuous) as the time covariate, (3) model with only the trimester (categorical) as the time covariate, where the variance-covariance pattern was assumed to be autoregressive, (4) model with both gestational age in weeks and continuous trimester as covariates, allowing random slope for gestational time in weeks, (5) model with both gestational age in weeks and continuous trimester as a covariate, allowing random slope for trimester, and (6) model with both gestational age in weeks and continuous trimester as a covariate, allowing random slopes for both gestational age in weeks and trimester. We then compared the six models and the unconditional mean model for each of the cytokines based on logic and statistical fitness, using Akaike information criteria (AIC) [16]. Because of the exploratory nature of this study, there was no correction for multiple comparisons.

## 3. Results

### 3.1. Detection Limits

The multiplex was designed to assess 42 factors, but the limits of detection were exceeded with three factors, and there were 5 cytokines that fell below the limit of detection in >50% of the samples. PDGF-AA, PDGF-AB/BB, and RANTES had cytokine levels above detection limits of the assay in 9.9%, 11.2%, and 34.9% of the samples, respectively (Table S1; see Supplementary Material available online at http://dx.doi.org/10.1155/2015/952571). IL-2, IL-3, IL-4, IL-13, and TNF*β* had >50% of the samples below the detection limit (Table S1).

### 3.2. Comparison between Pregnant and Nonpregnant Samples

**Figure 1:**
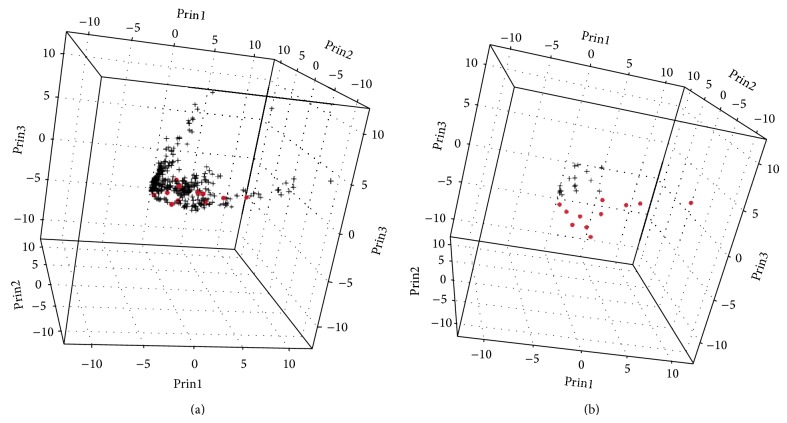
Principal components analysis of individual samples grouped by “pregnant” (+) versus “nonpregnant” (•). In (a, left), all samples are plotted with no clear separation of the groups. When only the baseline first trimester samples are plotted against the nonpregnant controls (b, right), a separation between the groups becomes apparent.

Differences in cytokine profiles between baseline (first trimester) pregnancy samples and nonpregnant control samples could be identified by PCA (Figure 1). Specifically, we identified significantly higher levels of GRO*α*, TGF*α*, EGF, PDGF-AA, and PDGF-AB/BB and significantly lower levels of sCD40L, IP-10, IL-6, IL-17, IL-13, and MCP-1 in the baseline pregnancy samples (Table 1). When all longitudinal samples were included in a PCA, no difference could otherwise be identified between sample groups. Next, we evaluated whether those differences apparent in early gestation were also present at the end of pregnancy. When comparing the final independent samples obtained at the end of pregnancy to the healthy control samples, the following remained significantly elevated in pregnant women: EGF (330.6 versus 24.8 pg/mL, *P* = 0.0013), GRO*α* (1,153.6 versus 341.6 pg/mL, *P* < 0.001), sIL-2Ra (17.9 pg/mL versus LLD, *P* = 0.02), and TGF*α* (15.8 versus 1.8 pg/mL, *P* = 0.0006). Meanwhile, only eotaxin was lower in women at the end of pregnancy compared to normal controls (33.4 versus 330.0 pg/mL, *P* < 0.001).

### 3.3. Longitudinal Analyses

**Figure 2:**
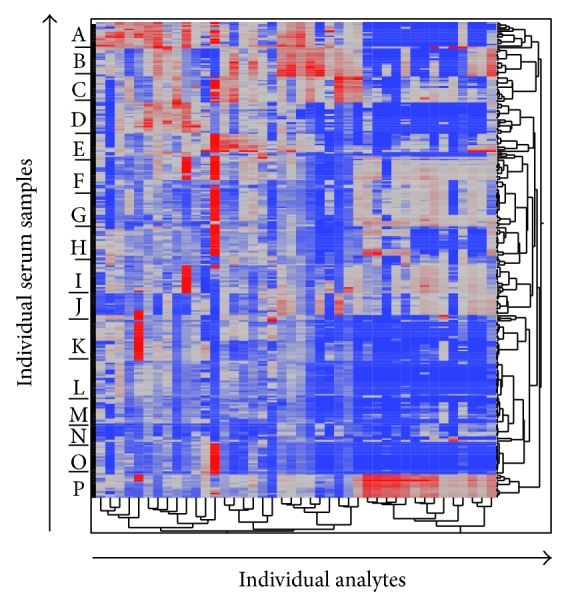
Unsupervised hierarchical clustering of all data. Protein levels of peripheral blood cytokines and growth factors as determined by multiplex cytokine array were log-normalized and clustered. Each row represents an individual serum sample, and each analyte measured with the multiplex cytokine array is represented by a column. Even with log-transformation, all subjects' samples exhibited a strong tendency to cluster with other samples from the same subject, giving the impression of distinct subject-specific “bands” (labeled A–P) within the cluster.

**Figure 3:**
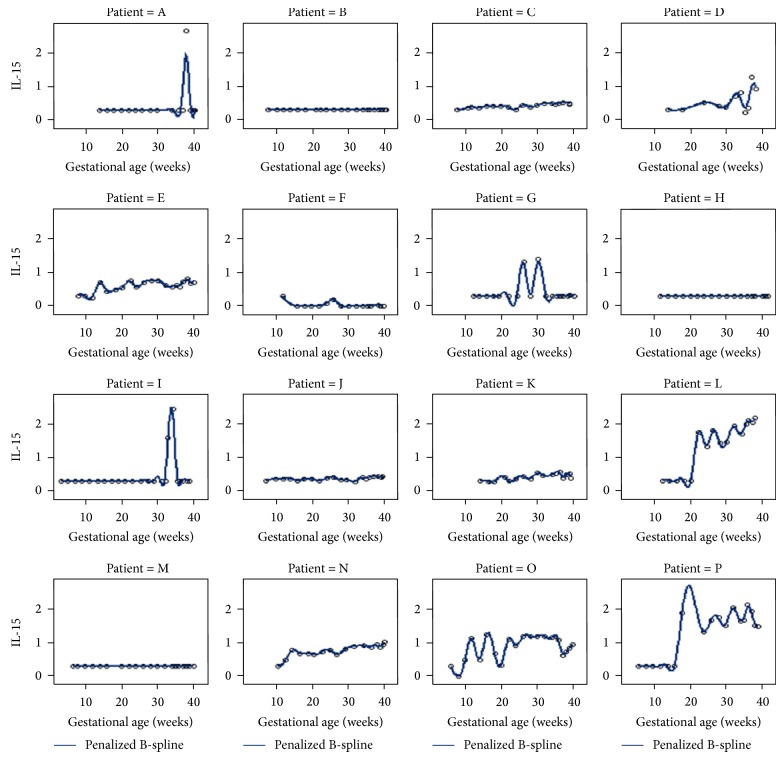
Nonparametric empirical growth trajectory of IL-15 using splines.

**Figure 4:**
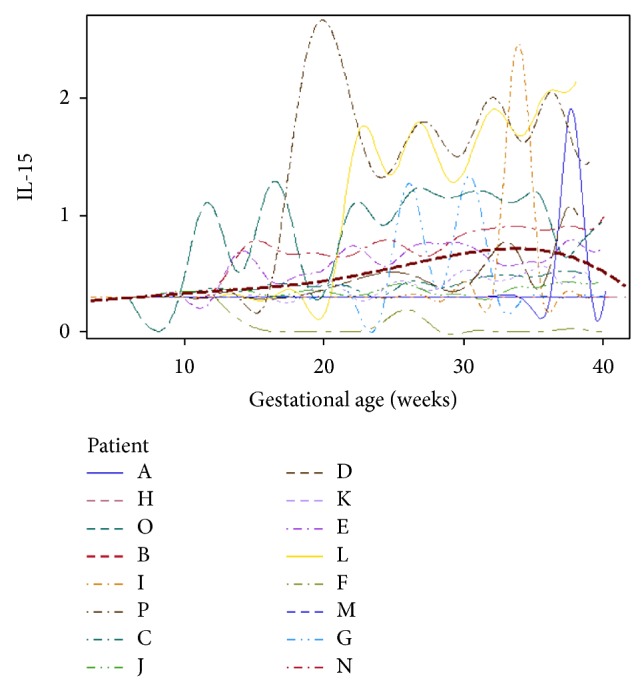
Growth curve of IL-15, with data from all patients superimposed, and with the average of all of the plots represented by the dashed red line.

To begin to assess within-subject variation, we clustered the raw data (Figure 2), where the issue of correlation within subjects becomes visually apparent. Subject-specific “bands” or cytokine fingerprints appear in the cluster map. Empirical growth plots demonstrated that the within-subject variation differs among different women (Figures 3 and 4 show a representative example using IL-15, and the remainder of the plots can be viewed in Supplementary Figures). In other words, some women display more variability than others. For example, patient O displays very large variation in the majority of the cytokines tested, whereas patients B, C, F, and G have comparatively steady levels for most cytokines. Also, high variability in one cytokine does not imply high variability in other cytokines for the individual women.

### 3.4. Unconditional Mean Models

High interclass correlation (≥0.7) was observed for sCD40L, RANTES, IL-10, TNF*β*, IL-7, and GM-CSF (Table S2). This means that the majority of the variability in these cytokines is attributed to variance between subjects. The interclass correlations were comparatively low (<0.3) for IL-3, IL-4, MCP-3, and IP-10, suggesting more variation within subjects for these cytokines.

#### 3.4.1. Cytokines with Gestational Weeks (GW) as Final Model

The final model for MDC (Table 2) was UGM with GW, with a decreasing trajectory. The estimated variance for the random slope is 0.000001632, suggesting that the variability of slope among patients is quite small. On the other hand, the average scatter of an individual's outcome around her own trajectory is larger (the estimated variance is 0.000937, *P* < 0.0001), suggesting that there might be some other important covariates that are not included in the model. However, including gestational age in weeks as a covariate does substantially improve the fit of the model. When comparing the estimated within-subject variance of the UGM with that from the UMM, we found that the linear gestational age in weeks helps to explain 34% of the within-subject variation in MDC. The estimated fixed effect suggests that MDC decreases over time (the estimated slope is −0.00163, with *P* ≤ 0.0001, suggesting that the slope is significantly lesser than 0, Figure S2). Eight other cytokines demonstrated statistical significance, although with increasing trajectories, with UGM and GW as a covariate: IL-1*β*, IL-6, IL-8, IL-12p70, IL-13, IL-15, IP-10, and FLT3-ligand (Table 2).

#### 3.4.2. Cytokines with Trimester as Final Model

Six cytokines showed association with trimester as a linear variable: IL-1ra, IL-3, IL-9, IL-12p40, IFN*α*2, and sCD40L (Table 3). IL-1ra showed the greatest percent explanation by inclusion of linear trimester in the model. When comparing the estimated within-subject variance of the UMM with that from the UGM, we found that the linear trimester helps to explain 27% of the within-subject variation in IL-1ra. Seven other cytokines showed significant associations with trimester as a categorical variable: IL-4, IFN*γ*, G-CSF, TGF-*α*, TNF-*β*, sIL-2ra, and MIP-1*α* (Table 4).

#### 3.4.3. No Change over Time

The final model for both EGF and VEGF was UMM with intercept only, leading to the conclusion that both EGF and VEGF do not change over time. Time-dependent UGM models were identified but did not meet statistical significance for the following cytokines: IL-2, IL-5, PDGF-AA, PDGF-AB, MIP-1*β*, GRO*α*, MCP-1, MCP-3, RANTES, IL-17, IL-7, eotaxin, FGF-2, IL-10, TNF*α*, IL-1ra2, fracktalkine, and GM-CSF. Therefore, approximately 50% of cytokines measured by multiplex array did not show any association with gestational week or trimester.

## 4. Discussion

Our results suggest that the third trimester of pregnancy is characterized by an increasingly inflammatory (e.g., IL-1*β*, IL-6, IL-12, IL-15, IP-10, and sCD40 ligand) as well as counterregulatory (e.g., IL-1ra and FLT3-ligand) milieu compared to earlier stages of pregnancy. Our superimposed growth curves of IL-15 (Figure 4) and IL-1*β* (Figure S3), the cytokines with the strongest association with gestational week as a linear covariate, suggest that this change begins at approximately week 20 and peaks just after week 30. This is consistent with other reports of systemic immune activation as well as counterregulation in the latter part of pregnancy [3, 17–19]. Increasing IL-1*β* and IL-15 may predominantly be related to production from placental tissues [20, 21] or mononuclear phagocytes [22, 23] in late gestation. The precise immunologic mechanisms responsible for this shift cannot be determined by our study, but if the source of these cytokines is predominantly the innate immune system, one potential stimulus to these cytokines' secretion is cell-free fetal DNA. A recently proposed model links rising circulating levels of fetal DNA to maternal innate immune activation via toll-like receptor- (TLR-) 9 on neutrophils and macrophages, leading to increasing inflammatory cytokine release, that may result in an immune cascade ultimately leading to parturition [24].

**Figure 5:**
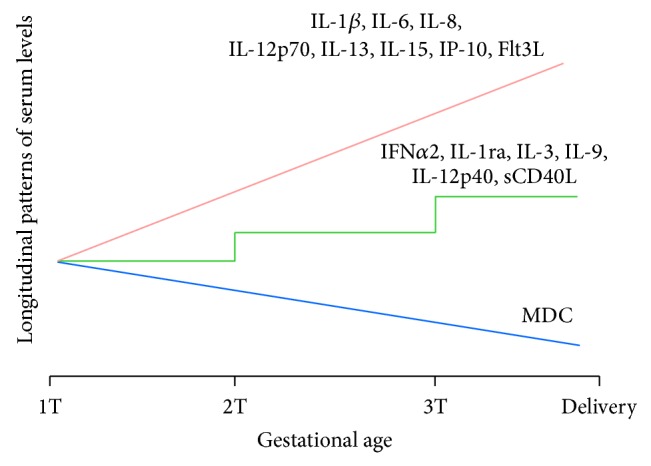
Cytokines with linear relationships to time-dependent variables.

A novel finding of this study is the decline in MDC levels throughout pregnancy (Figure 5). MDC is chemoattractant for immature dendritic cells [25] and type 2-biased T cells [26], and it is possible that decreasing MDC levels may reflect a gradual shift away from a self-amplifying type 2 immune response as pregnancy progresses. Another novel finding of this study is the increasing levels of IL-9 per trimester. IL-9 is cytokine that has been recognized for decades but only recently had its cell of origin identified: the innate lymphoid cell (ILC) [27]. The role of IL-9, which has historically been categorized as a Th2 cytokine prior to the recognition of ILC [28], is currently unknown in normal pregnancy. Because only serum was available for analysis, we cannot confirm the cellular source of the changes observed with this cohort. Nonetheless, increasing transcription levels of several innate immune components, including* CD14*, multiple* TLR* genes, and* IL-1B*, have been observed in peripheral blood leukocytes of women in the third trimester [19], suggesting that the changes in concentrations of the cytokines identified in our study could plausibly be due to changes in peripheral blood cellular composition. Further studies will be necessary to determine whether placental tissues, circulating leukocytes, stromal cells, or other sources also contribute to the changes reflected in our results.

Although these results show statistical significance, it is also noteworthy that the slopes of the variance were all fairly small for each of the analytes and that gestational week did not explain any more than 40% of the variation observed in any of the cytokines. Pregnancy clearly alters the maternal immune milieu, but the changes from baseline in the setting of normal pregnancy are subtle and more variable in some women than in others. What clinical factors contribute to this variation observed between women in the setting of a normal first pregnancy is not entirely known. Blood draws and resulting data were relatively sparse in the first trimester due to women not returning as frequently for study blood draws, even though they were scheduled per protocol, relative to the second and third trimesters. It is also important to note that many analytes were not detectable in samples; therefore, it is possible that a different method besides multiplex array on serum or plasma, for example, ELISA for individual cytokines/growth factors, may be more sensitive to detect change over time.

Previous longitudinal studies of maternal cytokines during pregnancy have shown some concordance with our results. Curry et al. investigated the change of maternal plasma cytokines from early to midgestation in a large cohort (approximately 1,000 patients) and found that IL-12 and IFN*γ* levels increased, while IL-2 and GM-CSF levels decreased as pregnancy progressed [1]. Our results confirmed increasing IL-12 levels, as well as an increase of IFN*γ* in the third trimester compared to the first. However, Kraus et al. published maternal multiplex ELISA results from 50 women tested at each trimester as well as postpartum and showed that IFN*γ* levels decreased throughout gestation [2]. Denney et al. also found that basal levels of IFN*γ* decreased throughout gestation, as did levels of TNF*α*, IL-1*β*, and IL-6 in serum samples of 45 healthy pregnant women collected during each trimester [3]. Another recent study demonstrated decreasing IL-1*β* levels throughout gestation [29]. While our study did not confirm any time-dependent change of TNF*α*, we instead observed increases in IL-1*β* and IL-6 throughout pregnancy, in contrast to these studies. Clearly, individual heterogeneity exists, and our smaller sample size may account for some of these differences with prior studies.

Although not the original focus of this study, we also identified several differences in cytokine/growth factor milieu when comparing pregnant women to healthy controls. Notably, the first trimester of pregnancy was characterized by an increase in several growth factors (GRO*α*, EGF, TGF*α*, and PDGF) and a relative decrease in inflammatory markers (sCD40L, IL-6, IL-17, IP-10, eotaxin, and MCP-1). Eotaxin, a potent chemoattractant for eosinophils during allergic reactions, has been similarly described to be suppressed during pregnancy by Kraus et al. [2]. Overall, the tolerance induction required for successful pregnancy at the level of the fetomaternal interface may also be observed in a relatively tolerogenic, growth factor-rich maternal systemic environment. The difference in sample source when comparing the archived samples from healthy pregnant women (serum) and normal controls (plasma) may contribute to these findings. For example, eotaxin, PDGF, EGF, VEGF, and soluble CD40 ligand have previously been shown to be significantly different in serum postclotting as compared to plasma samples [30]. Because of this potential source of variation in comparing our historical to contemporary samples, our results will require validation with similarly processed samples of the same source. However, EGF levels have been reported to be approximately 65 pg/mL in serum of healthy individuals [30], well below our median 489.4 pg/mL in our samples obtained from pregnant subjects, suggesting that our results may hold true despite differences in sample source. Similarly, serum levels of CD40L have been shown to be 8–30-fold higher in serum as compared to plasma [30], suggesting again that the very low levels identified in the serum of our cohort of pregnant women may indeed be reflective of the pregnancy state.

Our study is unique in that the longitudinal samples were obtained frequently throughout the course of pregnancy. Because of this, our study has the potential to more richly describe individual maternal immune variation over time. However, our study is limited by the relatively small sample size, the lack of pre- or postpartum samples, and potentially the age of the specimens. To address these limitations, we have recently conducted a larger longitudinal study of maternal immune changes during pregnancy, with peripheral blood samples obtained monthly throughout gestation as well as 6 weeks postpartum. Analysis of the clinical and laboratory data from our most recent study is ongoing.

Tolerance at the fetomaternal interface is complex and technically challenging to study in humans longitudinally. As a result, many recent studies have sought to understand how pregnancy changes maternal systemic immunity. The possibility exists of maternal peripheral blood not completely or accurately reflecting the local changes within decidual tissues. An example of such a discrepancy in our study is that of IP-10, which was low in first-trimester pregnancy compared to healthy controls in this cohort. IP-10 is secreted by decidual natural killer cells and is key to trophoblast migration and thus has previously been shown to be elevated during pregnancy [31, 32]. On the other hand, the decidual T cell compartment at parturition has recently been shown to closely reflect that of peripheral blood, suggesting that for some components of the immune system the maternal peripheral blood is reasonable to study [33]. Furthermore, sampling maternal peripheral blood is much more feasible to perform longitudinally as compared to more invasive means and may still lend insight into the maternal adaptation to pregnancy.

## 5. Conclusions

Successful pregnancy has previously been described as predominantly a type 2 immune response-biased phenomenon [34–36]. In light of the collective evidence, it seems that the description of pregnancy in terms of type 2 immune responses may be oversimplified, both in the overall description of key cytokines/growth factors as well as in the temporal dynamics, and it is in need of further refinement.

## Supplementary Material

Supplemental material accompanying this article incluldes detailed results of the number of samples with analyte concentrations below or above detection limits, interclass correlation coefficients for each cytokine, nonparametric empiric growth trajectories of individual cytokines with plots separated by individual patients, and the superimposed growth curves of MDC and IL-1b.

## Figures and Tables

**Table 1:** Comparison of protein concentrations (pg/mL) of growth factors and cytokines between controls and baseline pregnancy samples.

	Healthy control (*N* = 11)			Pregnancy baseline (*N* = 16)			
	Median	Min.	Max.	Median	Min.	Max.	*P*
Higher in pregnancy							
GRO*α*	341.6	243.3	742.6	1018.6	424.4	3208	<0.001
PDGF-AA	563.8	340.9	923.7	10740.6	2181	>ULD	<0.001
TGF*α*	1.8	<LLD	33.3	11.7	2.7	78.4	0.002
EGF	24.8	8.6	178.2	489.4	14.6	1506	0.003
PDGF-AB/BB	3410.4	493	7482	10760.7	3650	>ULD	0.005
Higher in controls							
Eotaxin	330.9	166.3	651	36.8	9.8	136.9	<0.001
sCD40L	424.7	132.7	1423	29.2	0	1583	0.004
IP-10	400.2	198.6	1271	162.7	57.8	380.7	0.009
IL-17	14.8	2.2	161.6	5.5	<LLD	46.9	0.03
IL-6	7.9	<LLD	82	<LLD	<LLD	34.3	0.03
IL-13	21.3	<LLD	95	<LLD	<LLD	37.3	0.05
MCP-1	492.6	176.9	1043	335.2	80.5	1002	0.05
Nonstatistically significant differences							
IL1-ra	81.4	<LLD	2087	21	<LLD	430.4	0.07
IFN*γ*	62.7	3.4	170	11.8	1.3	221.6	0.08
IL-12p70	5.7	<LLD	48.6	0.6	<LLD	18.9	0.09
IL-2	5	<LLD	50.8	0.1	<LLD	45.7	0.1
sIL2-ra	<LLD	<LLD	52.1	7.2	<LLD	71.7	0.1
VEGF	476.4	5.7	1399	189.5	<LLD	466.1	0.1
IL-12p40	48.6	<LLD	155.7	2.4	<LLD	333.8	0.2
IL-3	<LLD	<LLD	5.1	<LLD	<LLD	1.4	0.2
IL-4	<LLD	<LLD	98.3	<LLD	<LLD	31.1	0.2
MCP-3	10.4	<LLD	43.8	15.9	<LLD	76.5	0.2
RANTES	6093.6	1590	>ULD	22264.8	1696	>ULD	0.2
IFN*α*2	47	<LLD	378.6	22.8	5	719.3	0.3
IL-5	0.3	<LLD	1.1	0.6	<LLD	1.8	0.3
IL-9	<LLD	<LLD	27	<LLD	<LLD	9.9	0.3
Fractalkine	106.6	<LLD	2157	96.8	<LLD	514.6	0.4
MIP-1*β*	45.1	<LLD	366	50.1	27.6	196.5	0.4
TNF*β*	0.2	<LLD	20.5	<LLD	<LLD	36.6	0.4
G-CSF	25.1	7.3	220.6	29.2	6.3	619.8	0.5
IL-7	<LLD	<LLD	29	1.3	<LLD	219.3	0.5
IL-8	12.8	4.8	45.2	10.8	1.4	22.8	0.5
FGF-2	28.8	11.8	103.5	24.7	5.9	75.1	0.6
GM-CSF	94.1	14.3	456.3	65.7	23.7	3337	0.6
Il-1*β*	<LLD	<LLD	25.2	<LLD	<LLD	7.1	0.6
FLT3-ligand	7	<LLD	84.1	<LLD	<LLD	72.8	0.7
IL-15	<LLD	<LLD	11.1	0.9	<LLD	12.1	0.8
IL-10	<LLD	<LLD	19.1	<LLD	<LLD	17.1	0.9
IL-1ra2	3	<LLD	34.4	<LLD	<LLD	48.2	0.9
MDC/CCL22	1460.4	676	4049	1440.8	239.1	3665	0.9
MIP-1*α*	58.9	11.6	129	51.1	15.3	289	0.9
TNF*α*	3.8	<LLD	35.9	6.4	0.4	97.7	0.9

EGF = epidermal growth factor, FGF-2 = fibroblast growth factor-2, G-CSF = granulocyte colony stimulating factor, GM-CSF = granulocyte macrophage colony stimulating factor, GRO-a = growth-related oncogene alpha, IFN = interferon, IL = interleukin, IL-1ra = IL-1 receptor antagonist, IP-10 = interferon-gamma induced protein 10, MCP = monocyte chemotactic protein, MDC = macrophage-derived chemokine, MIP = macrophage inflammatory protein, PDGF = platelet-derived growth factor, RANTES = regulated on activation, normal T cell expressed and secreted, sCD40L = soluble CD40 ligand, sIL2-ra = soluble IL-2 receptor alpha, TGF-a = transforming growth factor alpha, TNF = tumor necrosis factor, and VEGF = vascular endothelial growth factor.

**Table 2:** Cytokines with gestational week (GW) as the time covariate, ordered by decreasing within-subject variance explained by adding GW to the unconditional growth model (UGM).

Analyte	Estimated random slope variance	Estimated scatter variance around individual's own trajectory	Within-subject variance explained by adding GW to UGM	Estimated fixed effect from GW	*P*
IL-15	0.000178	0.07753	0.39	0.01338	0.0004
IL-1*β*	0.000209	0.08174	0.38	0.01543	0.0001
MDC/CCL22	0.000001632	0.000937	0.34	−0.00163	*P* < 0.0001
IL-12p70	0.000159	0.11	0.34	0.00888	0.0174
FLT3-ligand	0.000518	0.243	0.32	0.01735	0.007
IP-10	0.000012	0.01545	0.31	0.00596	*P* < 0.0001
IL-13	0.000144	0.07619	0.31	0.008165	0.0176
IL-6	0.000325	0.1921	0.26	0.01603	0.0022
IL-8	0.00003	0.02932	0.11	0.00527	0.0023

**Table 3:** Cytokines with linear trimester as the time covariate, ordered by decreasing within-subject variance explained by adding trimester to the unconditional growth model (UGM).

Analyte	Estimated random slope variance	Estimated scatter variance around individual's own trajectory	Within-subject variance explained by adding trimester to UGM	Estimated fixed effect from trimester	*P*
IL-1ra	0.04697	0.2513	0.27	0.1872	0.0054
IL-9	0.02779	0.1134	0.25	0.1618	0.0011
IL-12p40	0.08139	0.1892	0.24	0.1592	0.0446
sCD40L	0.09832	0.2156	0.14	0.2178	0.0122
IFN*α*2	0.002279	0.01625	0.20	0.05875	0.0002
IL-3	0.006149	0.06967	0.10	0.06972	0.0146

**Table 4:** Cytokines with categorical trimester as the time covariate.

Analyte	Trim.	Estimate	Std. error	DF	*t* value	Pr > |*t*|
G-CSF						
Intercept		0.5324	0.0427	15	12.47	<0.0001
Trim.	1	−0.1757	0.03662	30	−4.8	<0.0001
Trim.	2	−0.0267	0.02718	30	−0.98	0.3334
Trim.	3	0	.	.	.	.
IFN*γ*						
Intercept		0.4027	0.02978	15	13.52	<0.0001
Trim.	1	−0.0837	0.03167	30	−2.64	0.013
Trim.	2	−0.0367	0.02397	30	−1.53	0.1361
Trim.	3	0	.	.	.	.
IL-4						
Intercept		0.5254	0.05158	15	10.19	<0.0001
Trim.	1	−0.1826	0.08129	30	−2.25	0.0322
Trim.	2	−0.2063	0.08505	30	−2.43	0.0215
Trim.	3	0	.	.	.	.
MIP-1*α*						
Intercept		0.334	0.01941	15	17.21	<0.0001
Trim.	1	−0.0369	0.01379	30	−2.67	0.012
Trim.	2	−0.018	0.01025	30	−1.75	0.0898
Trim.	3	0	.	.	.	.
sIL2Ra						
Intercept		0.8667	0.2036	15	4.26	0.0007
Trim.	1	−0.3954	0.186	30	−2.13	0.0418
Trim.	2	−0.184	0.1386	30	−1.33	0.1945
Trim.	3	0	.	.	.	.
TGF*α*						
Intercept		0.3781	0.04092	15	9.24	<0.0001
Trim.	1	−0.0498	0.02372	30	−2.1	0.0442
Trim.	2	−0.0155	0.01805	30	−0.86	0.3977
Trim.	3	0	.	.	.	.
TNF*β*						
Intercept		0.4825	0.09447	15	5.11	0.0001
Trim.	1	−0.1327	0.04807	30	−2.76	0.0097
Trim.	2	−0.0461	0.03632	30	−1.27	0.2147
Trim.	3	0	.	.	.	.

## References

[1] Curry A. E., Vogel I., Skogstrand K. (2008). Maternal plasma cytokines in early- and mid-gestation of normal human pregnancy and their association with maternal factors. *Journal of Reproductive Immunology*.

[2] Kraus T. A., Sperling R. S., Engel S. M. (2010). Peripheral blood cytokine profiling during pregnancy and post-partum periods. *The American Journal of Reproductive Immunology*.

[3] Denney J. M., Nelson E. L., Wadhwa P. D. (2011). Longitudinal modulation of immune system cytokine profile during pregnancy. *Cytokine*.

[4] Jamieson D. J., Honein M. A., Rasmussen S. A. (2009). H1N1 2009 influenza virus infection during pregnancy in the USA. *The Lancet*.

[5] Getahun D., Fassett M. J., Longstreth G. F. (2014). Association between maternal inflammatory bowel disease and adverse perinatal outcomes. *Journal of Perinatology*.

[6] Curry A. E., Vogel I., Drews C. (2007). Mid-pregnancy maternal plasma levels of interleukin 2, 6, and 12, tumor necrosis factor-alpha, interferon-gamma, and granulocyte-macrophage colony-stimulating factor and spontaneous preterm delivery. *Acta Obstetricia et Gynecologica Scandinavica*.

[7] Gargano J. W., Holzman C., Senagore P. (2008). Mid-pregnancy circulating cytokine levels, histologic chorioamnionitis and spontaneous preterm birth. *Journal of Reproductive Immunology*.

[8] Tsiartas P., Holst R. M., Wennerholm U. B. (2012). Prediction of spontaneous preterm delivery in women with threatened preterm labour: a prospective cohort study of multiple proteins in maternal serum. *British Journal of Obstetrics and Gynaecology*.

[9] Brewster J. A., Orsi N. M., Gopichandran N., McShane P., Ekbote U. V., Walker J. J. (2008). Gestational effects on host inflammatory response in normal and pre-eclamptic pregnancies. *European Journal of Obstetrics Gynecology and Reproductive Biology*.

[10] Molvarec A., Szarka A., Walentin S. (2011). Serum leptin levels in relation to circulating cytokines, chemokines, adhesion molecules and angiogenic factors in normal pregnancy and preeclampsia. *Reproductive Biology and Endocrinology*.

[11] Unal E. R., Cierny J. T., Roedner C., Newman R., Goetzl L. (2011). Maternal inflammation in spontaneous term labor. *American Journal of Obstetrics & Gynecology*.

[12] de Jager W., Bourcier K., Rijkers G. T., Prakken B. J., Seyfert-Margolis V. (2009). Prerequisites for cytokine measurements in clinical trials with multiplex immunoassays. *BMC Immunology*.

[13] Hornung R. W., Reed L. D. (1990). Estimation of average concentration in the presence of nondetectable values. *Applied Occupational and Environmental Hygiene*.

[14] Patil R., Shukre S., Paranjape R., Thakar M. (2013). Heparin and EDTA anticoagulants differentially affect the plasma cytokine levels in humans. *Scandinavian Journal of Clinical and Laboratory Investigation*.

[15] Ward J. H. (1963). Hierarchical grouping to optimize an objective function. *Journal of the American Statistical Association*.

[16] Akaike H. (1973). Information theory and an extension of the maximum likelihood principle. *Proceedings of the 2nd International Symposium on Information Theory*.

[17] Sacks G. P., Studena K., Sargent I. L., Redman C. W. G. (1998). Normal pregnancy and preeclampsia both produce inflammatory changes in peripheral blood leukocytes akin to those of sepsis. *The American Journal of Obstetrics and Gynecology*.

[18] Germain S. J., Sacks G. P., Sooranna S. R., Sargent I. L., Redman C. W. (2007). Systemic inflammatory priming in normal pregnancy and preeclampsia: the role of circulating syncytiotrophoblast microparticles. *The Journal of Immunology*.

[19] Jiang X., Bar H. Y., Yan J. (2012). Pregnancy induces transcriptional activation of the peripheral innate immune system and increases oxidative DNA damage among healthy third trimester pregnant women. *PLoS ONE*.

[20] Agarwal R., Loganath A., Roy A. C., Wong Y. C., Ng S. C. (2001). Expression profiles of interleukin-15 in early and late gestational human placenta and in pre-eclamptic placenta. *Molecular Human Reproduction*.

[21] Elliott C. L., Loudon J. A. Z., Brown N., Slater D. M., Bennett P. R., Sullivan M. H. F. (2001). IL-1*β* and IL-8 in human fetal membranes: changes with gestational age, labor, and culture conditions. *American Journal of Reproductive Immunology*.

[22] Grabstein K. H., Eisenman J., Shanebeck K. (1994). Cloning of a T cell growth factor that interacts with the *β* chain of the interleukin-2 receptor. *Science*.

[23] Hogquist K. A., Unanue E. R., Chaplin D. D. (1991). Release of IL-1 from mononuclear phagocytes. *Journal of Immunology*.

[24] Phillippe M. (2014). Cell-free fetal DNA—a trigger for parturition. *The New England Journal of Medicine*.

[25] Godiska R., Chantry D., Raport C. J. (1997). Human macrophage-derived chemokine (MDC), a novel chemoattractant for monocytes, monocyte-derived dendritic cells, and natural killer cells. *Journal of Experimental Medicine*.

[26] Andrew D. P., Chang M.-S., McNinch J. (1998). STCP-1 (MDC) CC chemokine acts specifically on chronically activated Th2 lymphocytes and is produced by monocytes on stimulation with Th2 cytokines IL-4 and IL-13. *Journal of Immunology*.

[27] Wilhelm C., Hirota K., Stieglitz B. (2011). An IL-9 fate reporter demonstrates the induction of an innate IL-9 response in lung inflammation. *Nature Immunology*.

[28] Demoulin J.-B., Renauld J.-C. (1998). Interleukin 9 and its receptor: an overview of structure and function. *International Reviews of Immunology*.

[29] Ferguson K. K., McElrath T. F., Chen Y.-H., Mukherjee B., Meeker J. D. (2014). Longitudinal profiling of inflammatory cytokines and C-reactive protein during uncomplicated and preterm pregnancy. *American Journal of Reproductive Immunology*.

[30] Biancotto A., Feng X., Langweiler M., Young N. S., Philip McCoy J. (2012). Effect of anticoagulants on multiplexed measurement of cytokine/chemokines in healthy subjects. *Cytokine*.

[31] Hanna J., Goldman-Wohl D., Hamani Y. (2006). Decidual NK cells regulate key developmental processes at the human fetal-maternal interface. *Nature Medicine*.

[32] Szarka A., Rigó J., Lázár L., Beko G., Molvarec A. (2010). Circulating cytokines, chemokines and adhesion molecules in normal pregnancy and preeclampsia determined by multiplex suspension array. *BMC Immunology*.

[33] Loewendorf A. I., Nguyen T. A., Yesayan M. N., Kahn D. A. (2014). Normal human pregnancy results in maternal immune activation in the periphery and at the uteroplacental interface. *PLoS ONE*.

[34] Wegmann T. G., Lin H., Guilbert L., Mosmann T. R. (1993). Bidirectional cytokine interactions in the maternal-fetal relationship: is successful pregnancy a TH2 phenomenon?. *Immunology Today*.

[35] Lin H., Mosmann T. R., Guilbert L., Tuntipopipat S., Wegmann T. G. (1993). Synthesis of T helper 2-type cytokines at the maternal-fetal interface. *The Journal of Immunology*.

[36] Doria A., Cutolo M., Ghirardello A. (2012). Effect of pregnancy on serum cytokines in SLE patients. *Arthritis Research and Therapy*.

