# Supplementary material for: Growth Modeling of the Maternal Cytokine Milieu throughout Normal Pregnancy: Macrophage-Derived Chemokine Decreases as Inflammation/Counterregulation Increases
Source: J Immunol Res. 2015 Mar 17;2015:952571. doi: 10.1155/2015/952571 (PMC4381731; doi:10.1155/2015/952571)

**Table S1. Number and percentages of the samples below or above limit of detection (LD) of the multiplex cytokine/growth factor assay (N=312 individual samples).**

|  | # below LD | # above LD | % below LD | % above LD |
| --- | --- | --- | --- | --- |
| EGF | 5 | 0 | 1.6% | 0.0% |
| Eotaxin | 2 | 0 | 0.6% | 0.0% |
| FGF-2 | 4 | 0 | 1.3% | 0.0% |
| Flt3 Ligand | 154 | 0 | 49.4% | 0.0% |
| Fracktalkine | 17 | 0 | 5.4% | 0.0% |
| G-CSF | 0 | 0 | 0.0% | 0.0% |
| GM-CSF | 0 | 0 | 0.0% | 0.0% |
| GRO | 0 | 0 | 0.0% | 0.0% |
| IFNα2 | 9 | 0 | 2.9% | 0.0% |
| IFN-γ | 3 | 0 | 1.0% | 0.0% |
| IL-10 | 142 | 0 | 45.5% | 0.0% |
| IL-12p40 | 114 | 0 | 36.5% | 0.0% |
| IL-12p70 | 149 | 0 | 47.8% | 0.0% |
| IL-13 | 160 | 0 | 51.3% | 0.0% |
| IL-15 | 146 | 0 | 46.8% | 0.0% |
| IL-17 | 12 | 0 | 3.8% | 0.0% |
| Il-1b | 155 | 0 | 49.7% | 0.0% |
| IL1-ra | 98 | 0 | 31.4% | 0.0% |
| IL-1ra2 | 155 | 0 | 49.7% | 0.0% |
| IL-2 | 161 | 0 | 51.6% | 0.0% |
| IL-3 | 224 | 0 | 71.8% | 0.0% |
| IL-4 | 212 | 0 | 67.9% | 0.0% |
| IL-5 | 46 | 0 | 14.7% | 0.0% |
| IL-6 | 117 | 0 | 37.5% | 0.0% |
| IL-7 | 135 | 0 | 43.3% | 0.0% |
| IL-8 | 0 | 0 | 0.0% | 0.0% |
| IL-9 | 135 | 0 | 43.3% | 0.0% |
| IP-10 | 0 | 0 | 0.0% | 0.0% |
| MCP-1 | 0 | 0 | 0.0% | 0.0% |
| MCP-3 | 52 | 0 | 16.7% | 0.0% |
| MDC | 0 | 0 | 0.0% | 0.0% |
| MIP-1α | 7 | 0 | 2.2% | 0.0% |
| MIP-1β | 6 | 0 | 1.9% | 0.0% |
| PDGF-AA | 0 | 31 | 0.0% | 9.9% |
| PDGF-AB/BB | 0 | 35 | 0.0% | 11.2% |
| RANTES | 0 | 109 | 0.0% | 34.9% |
| sCD40L | 0 | 0 | 0.0% | 0.0% |
| sIL2-ra | 1 | 0 | 0.3% | 0.0% |
| TGFα | 8 | 0 | 2.6% | 0.0% |
| TNFα | 1 | 0 | 0.3% | 0.0% |
| TNFβ | 163 | 0 | 52.2% | 0.0% |
| VEGF | 7 | 0 | 2.2% | 0.0% |

**Table S2. Interclass correlation coefficients for each cytokine.**

| **Analyte** | **Intraclass correlation coefficient (between subject variance)** |
| --- | --- |
| GM-CSF | 0.76 |
| TNF-b | 0.73 |
| IL-7 | 0.73 |
| IL-10 | 0.71 |
| RANTES | 0.71 |
| sCD40L | 0.7 |
| s-IL-2RA | 0.7 |
| Fracktalkine | 0.68 |
| VEGF | 0.68 |
| TGF-a | 0.66 |
| MIP1a | 0.65 |
| IL-12p40 | 0.64 |
| IL-1ra2 | 0.63 |
| IL-17 | 0.61 |
| Eotaxin | 0.61 |
| MIP1b | 0.58 |
| EGF | 0.58 |
| G-CSF | 0.57 |
| TNF-a | 0.55 |
| IL-1b | 0.5 |
| IL-9 | 0.5 |
| FLT3 ligand | 0.47 |
| MCP-1 | 0.46 |
| IL-6 | 0.45 |
| IL-15 | 0.45 |
| MDC (CCL22) | 0.44 |
| IL-8 | 0.41 |
| IL-13 | 0.41 |
| FGF-2 | 0.39 |
| IFNg | 0.38 |
| PDGF-AA | 0.37 |
| IL-1ra | 0.37 |
| GROa | 0.36 |
| IFNa2 | 0.36 |
| IL-5 | 0.33 |
| IL-2 | 0.33 |
| IL-12p70 | 0.32 |
| PDGF-AB/BB | 0.31 |
| IL-3 | 0.29 |
| IP-10 | 0.23 |
| MCP-3 | 0.22 |
| IL-4 | 0.1 |

**Figure S1. Nonparametric empirical growth trajectory using splines, with plots separated by individual patient.**

1. EGF


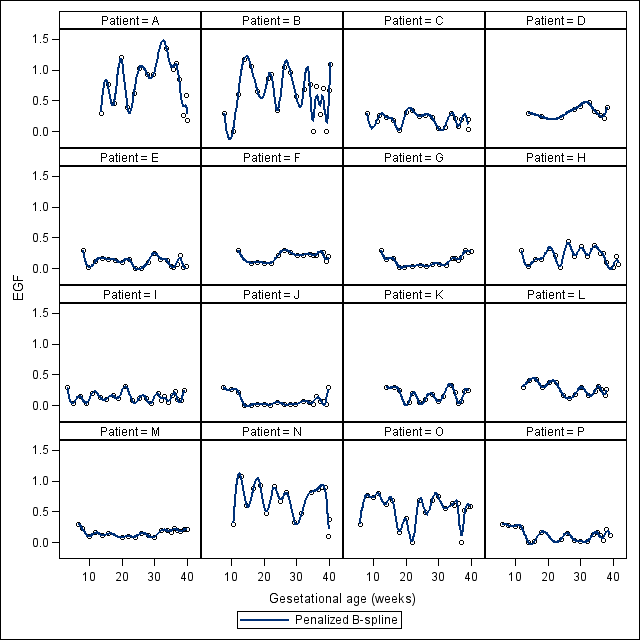


1. Eotaxin


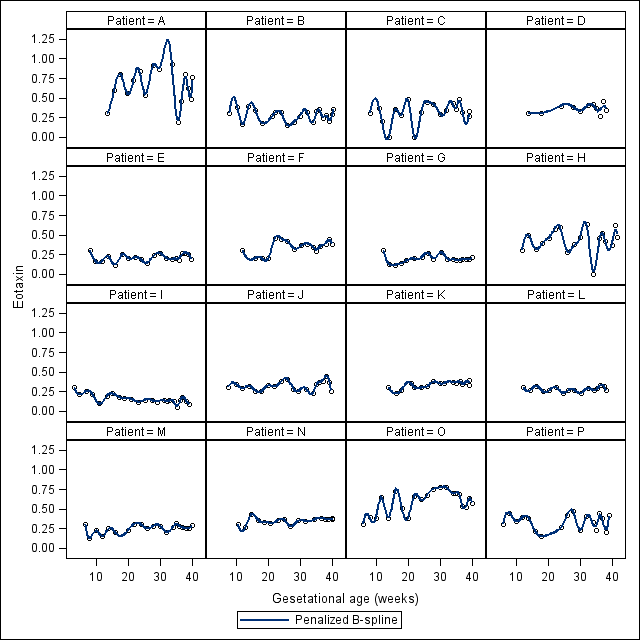


1. FGF-2


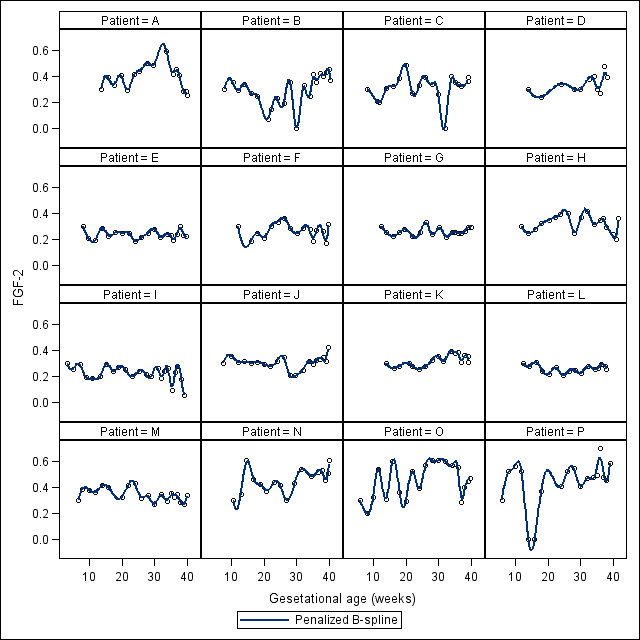


1. FLT-3 Ligand


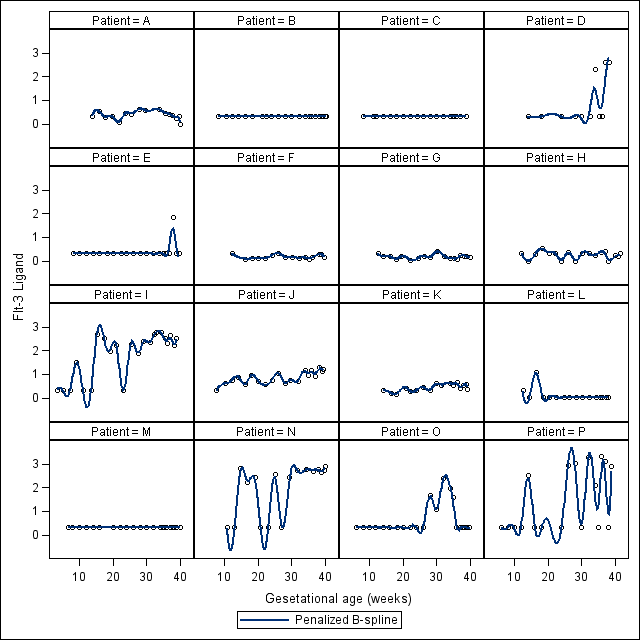


1. Fractalkine


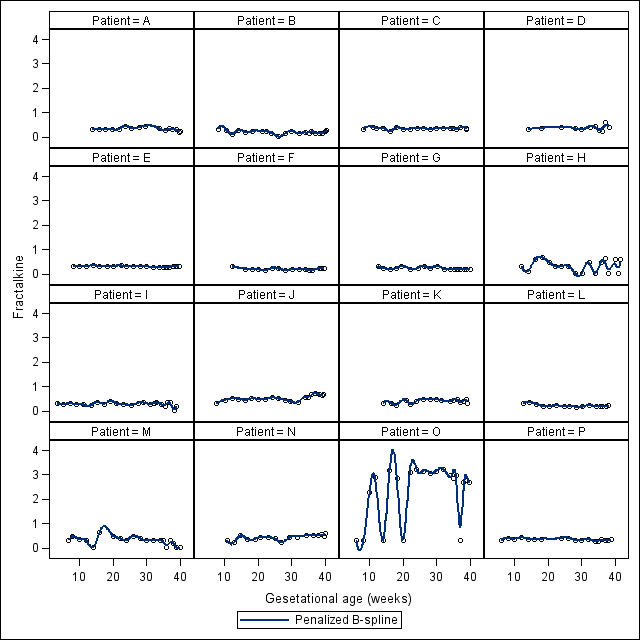


1. G-CSF


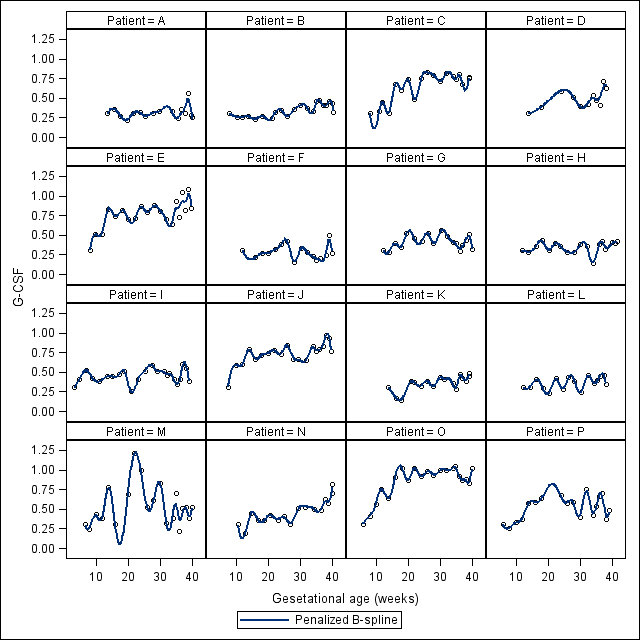


1. GM-CSF


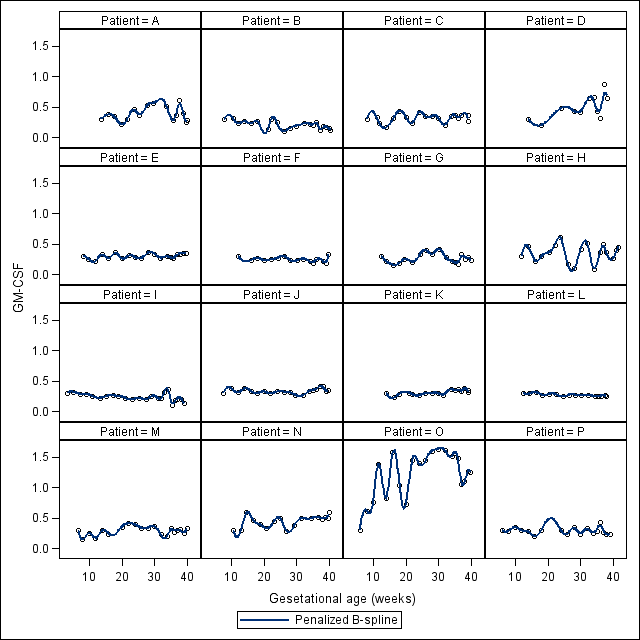


1. GRO


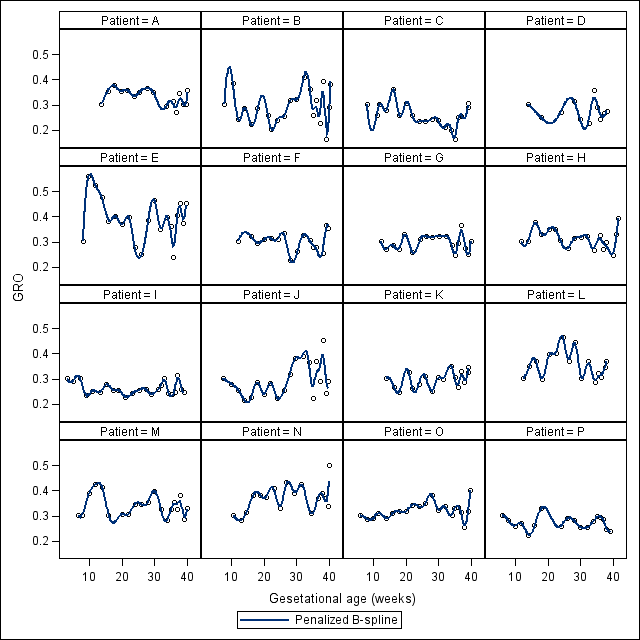


1. IFNα2


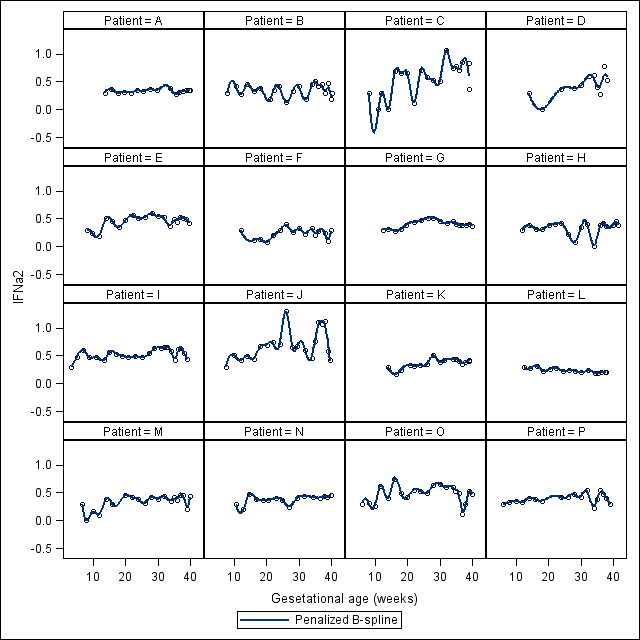


1. IFN-γ


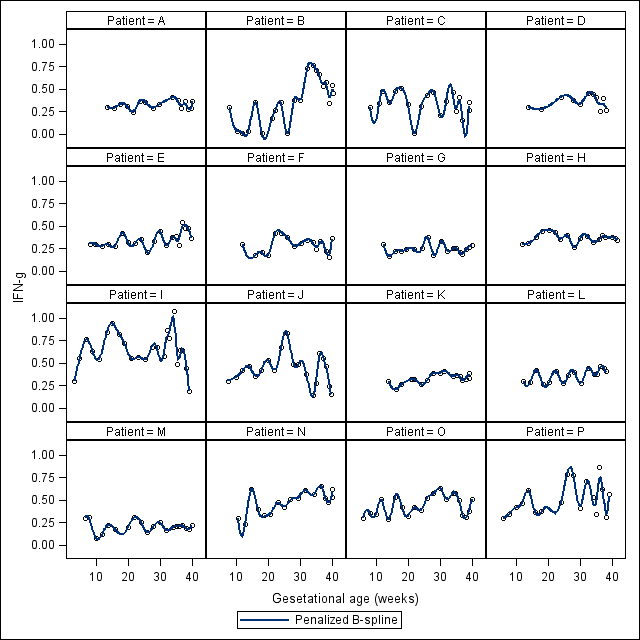


1. IL-1ra


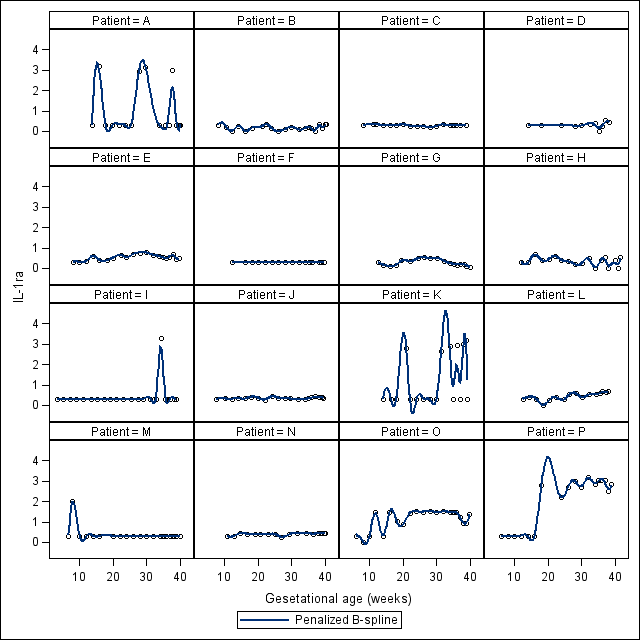


1. IL-1β


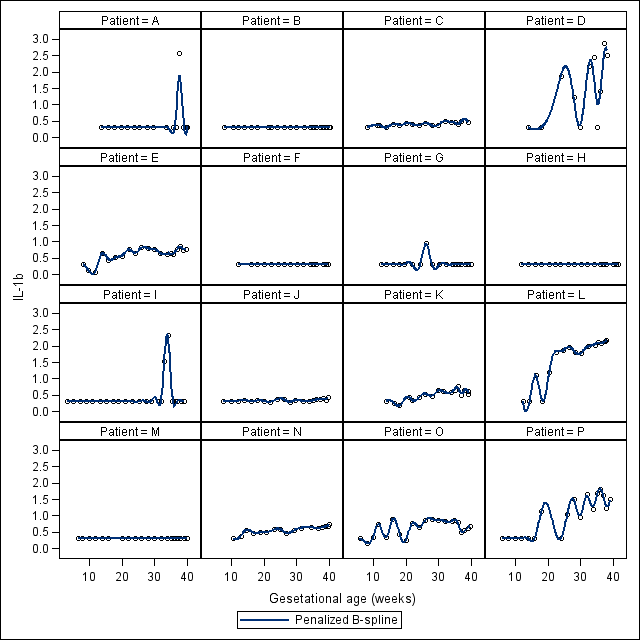


1. IL-1ra2


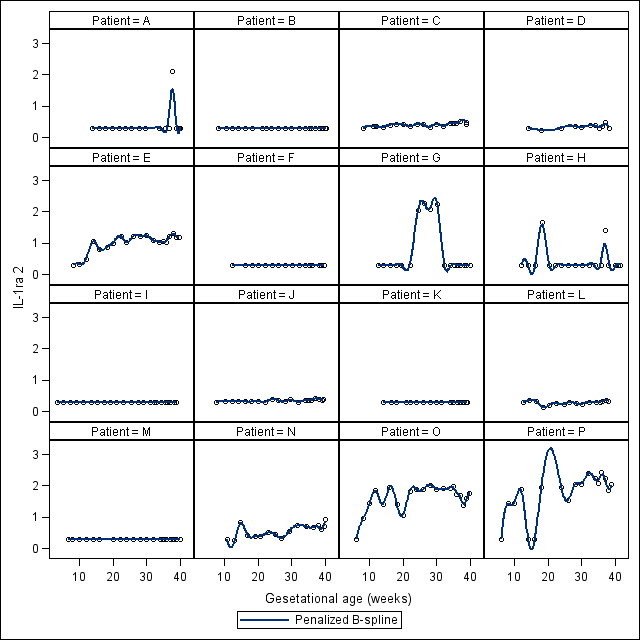


1. IL-2


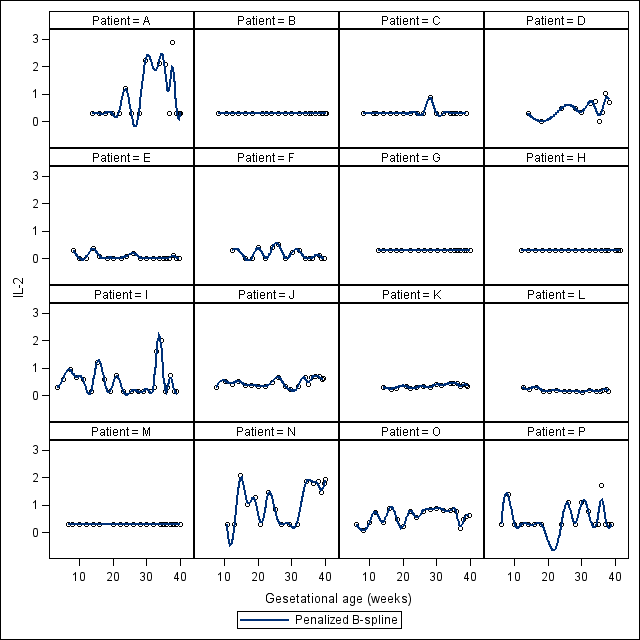


1. IL-3


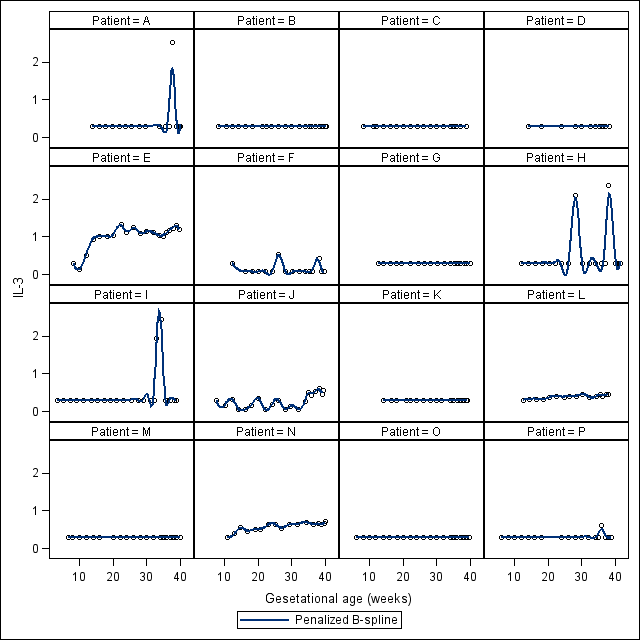


1. IL-4


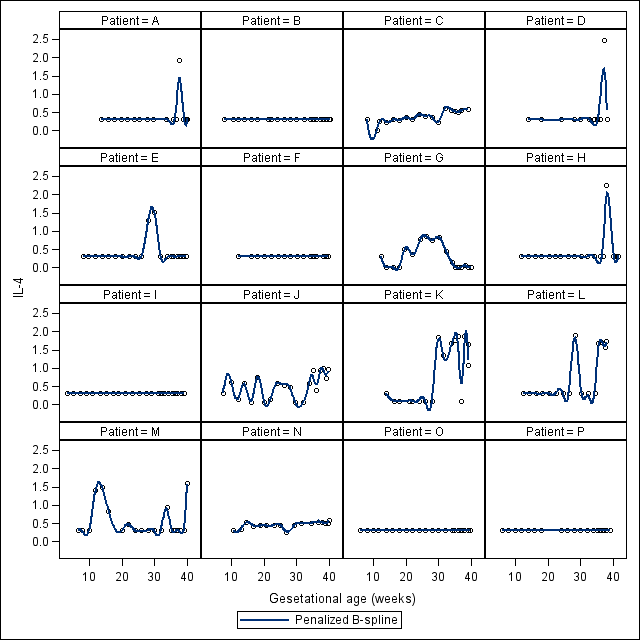


1. IL-5


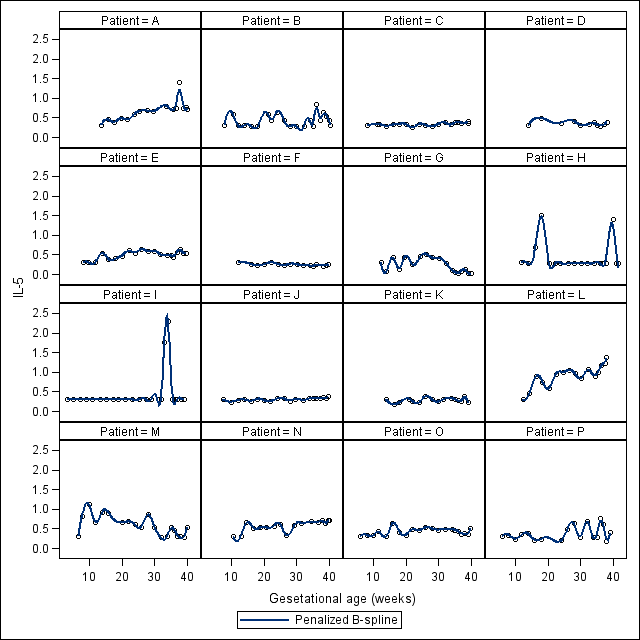


1. IL-6


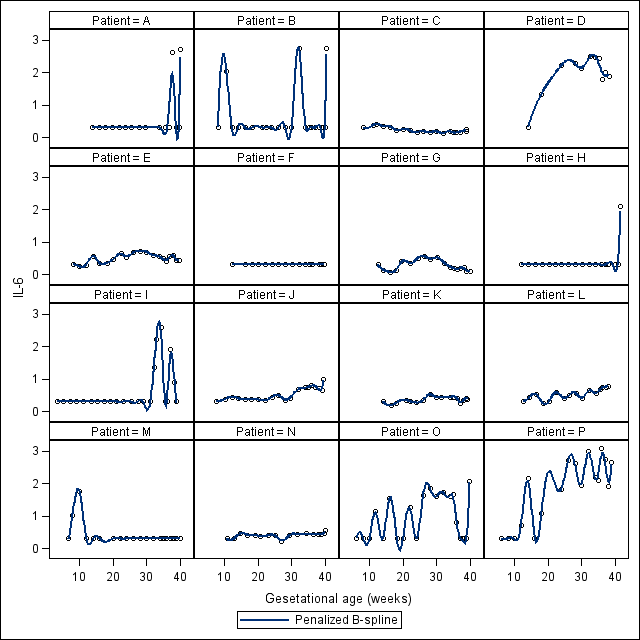


1. IL-7


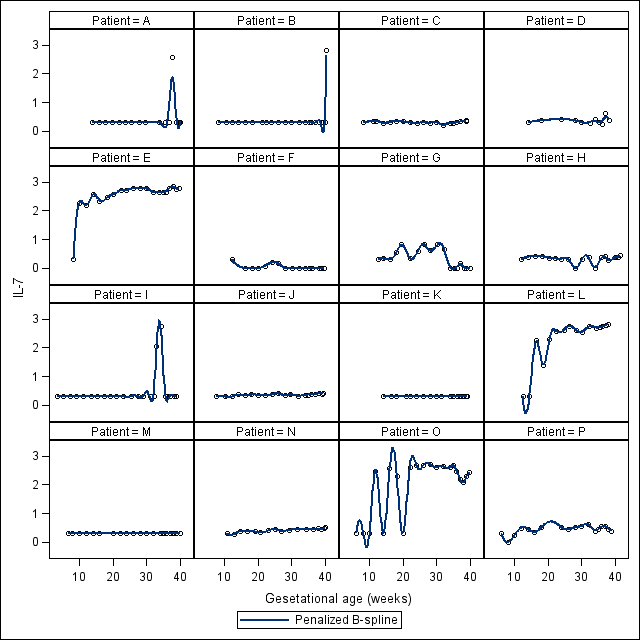


1. IL-8


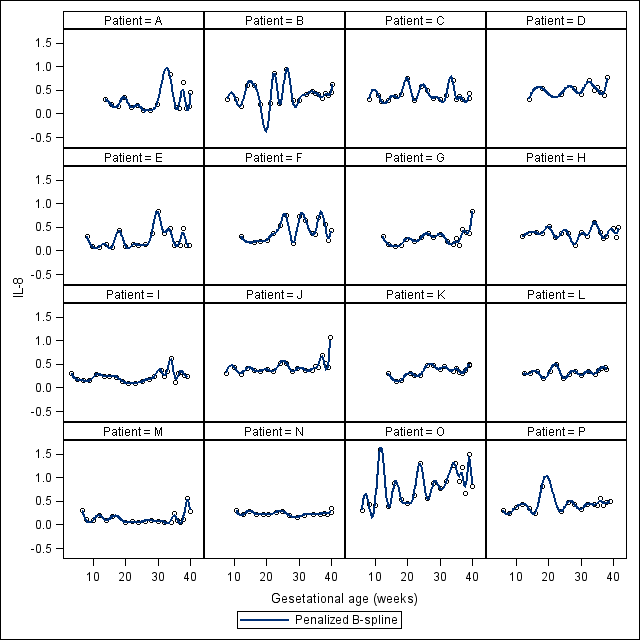


1. IL-9


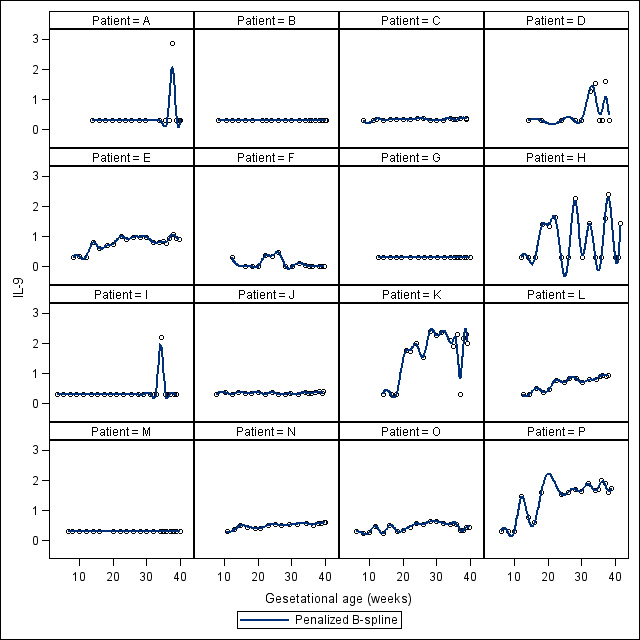


1. IL-10


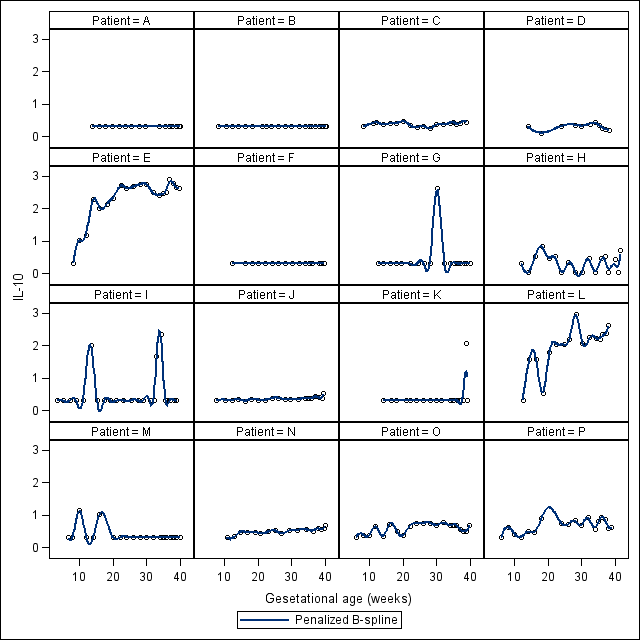


1. IL-12p40


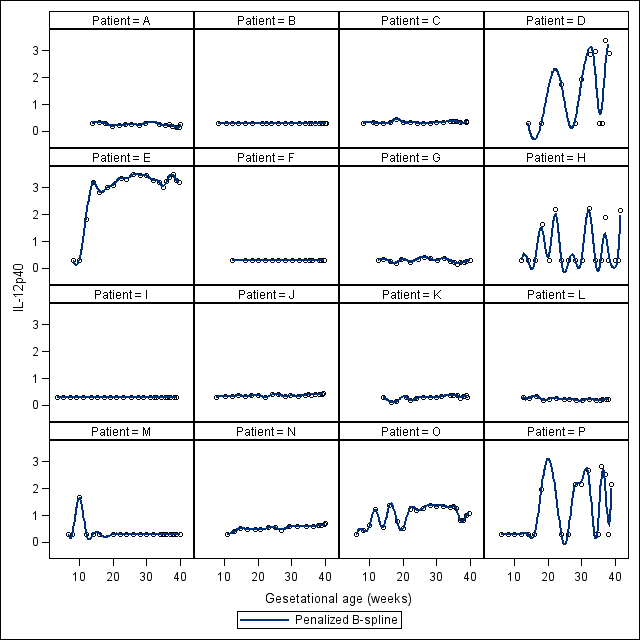


1. IL-12p70


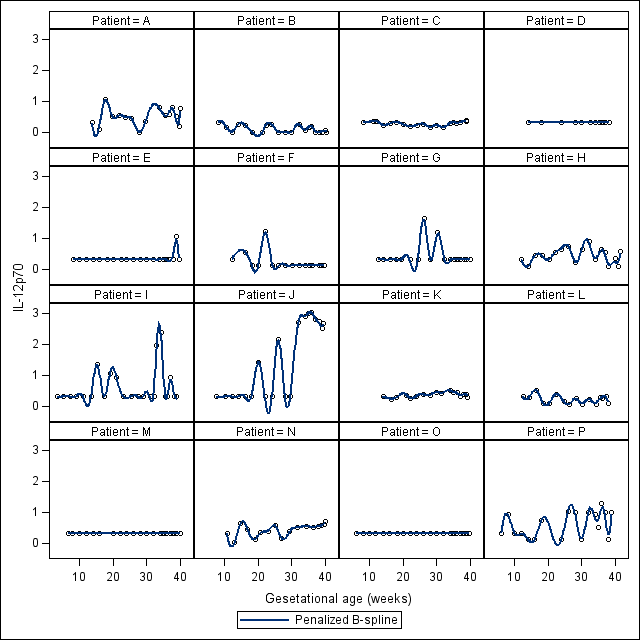


1. IL-13


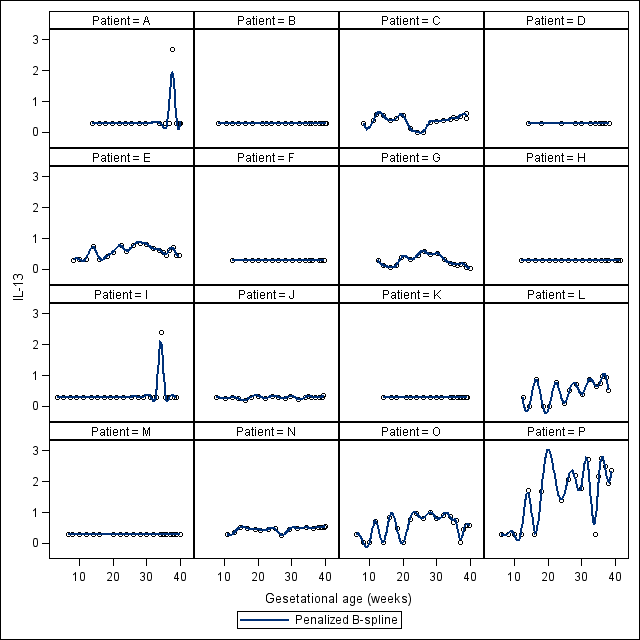


1. IL-15


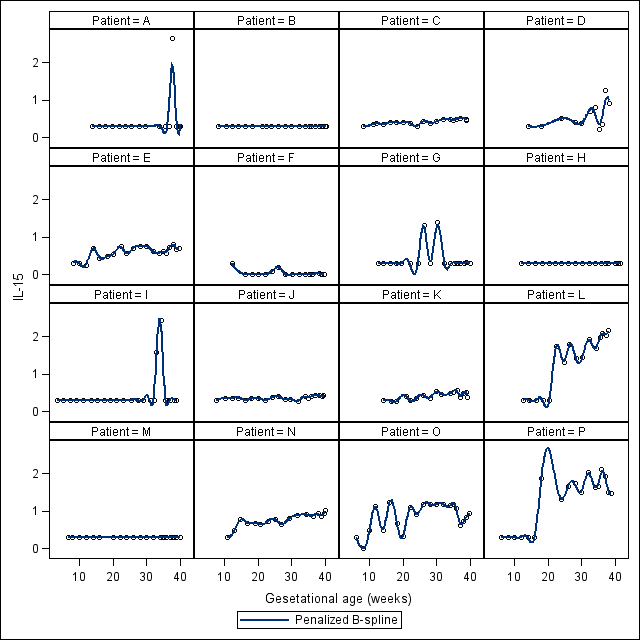


1. IL-17


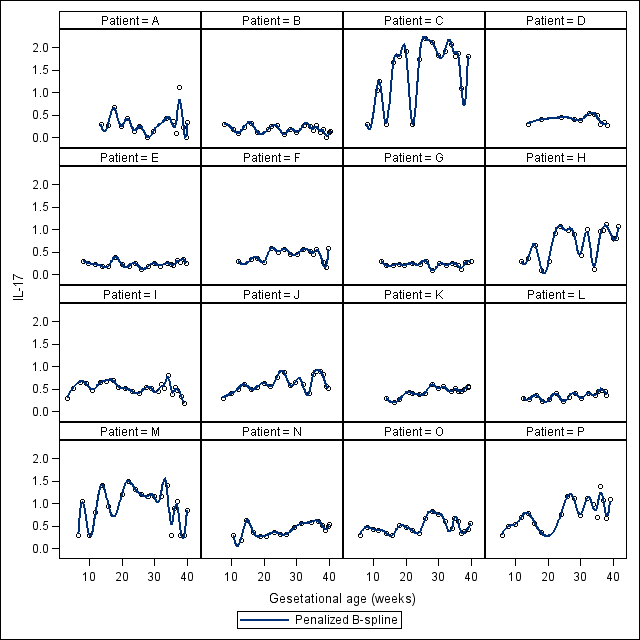


1. IP-10


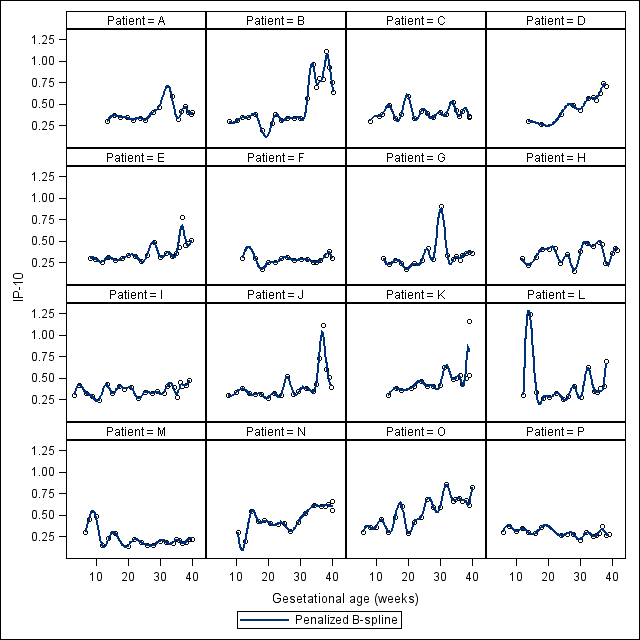


1. MCP-1


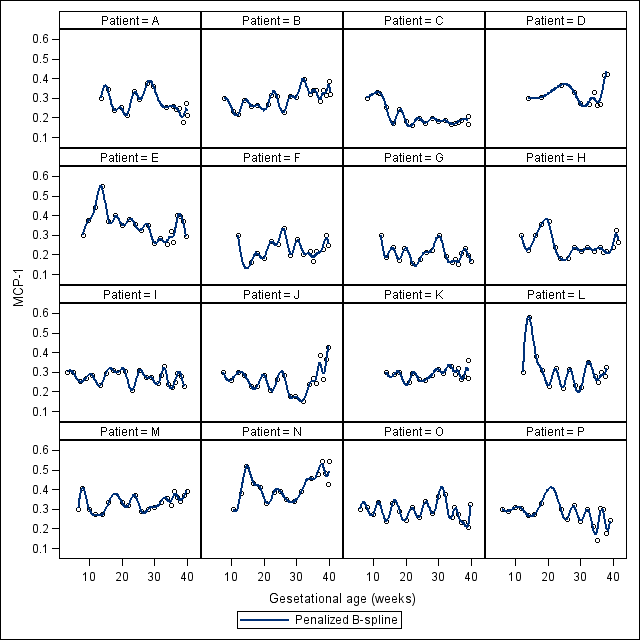


1. MCP-3


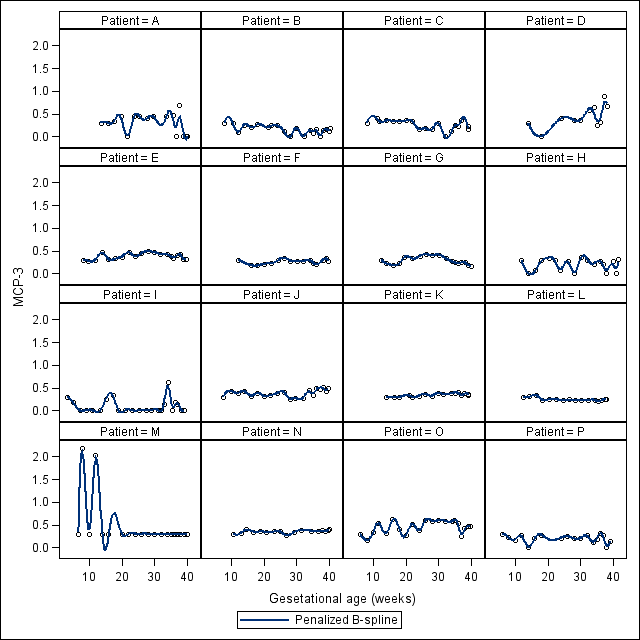


1. MDC


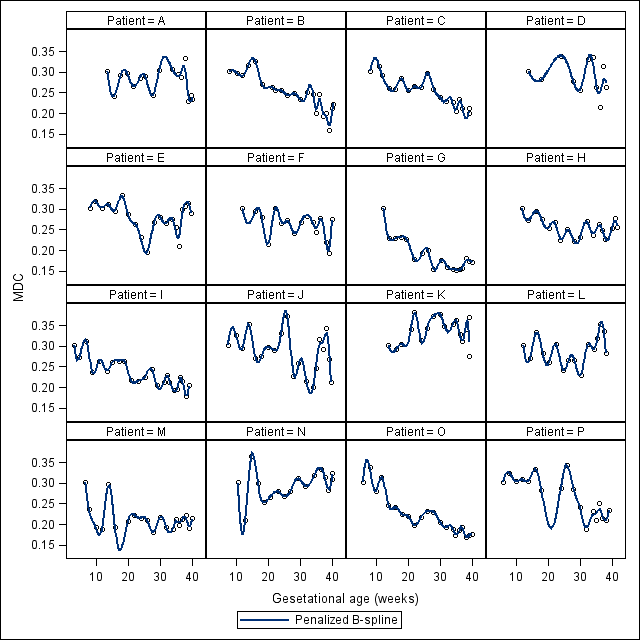


1. MIP-1α


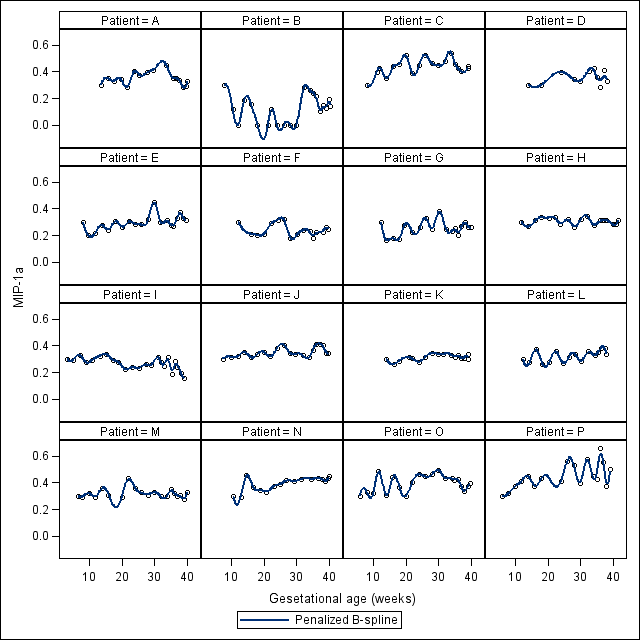


1. MIP-1β


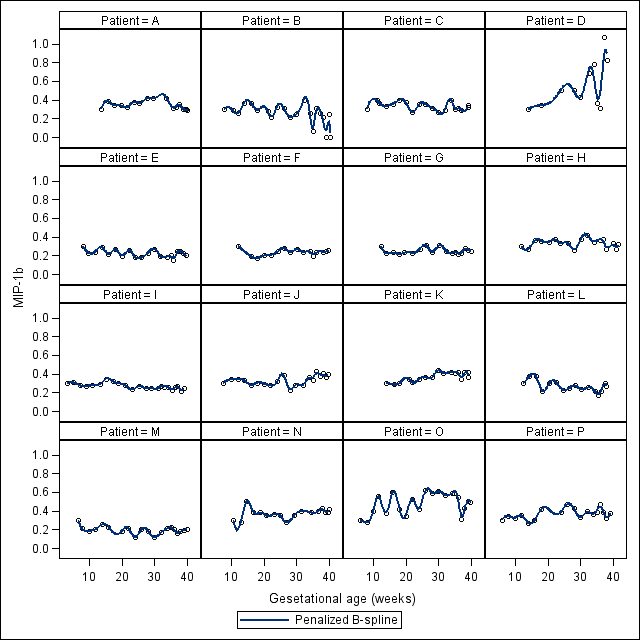


1. PDGF-AA


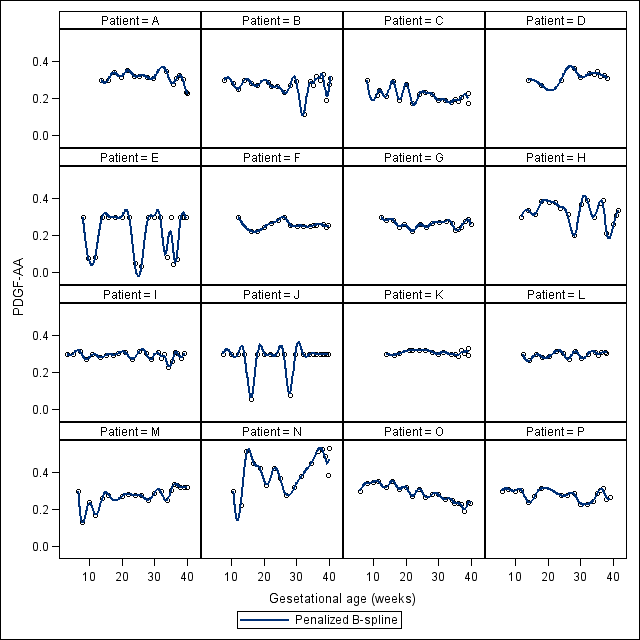


1. PDGF-AB/BB


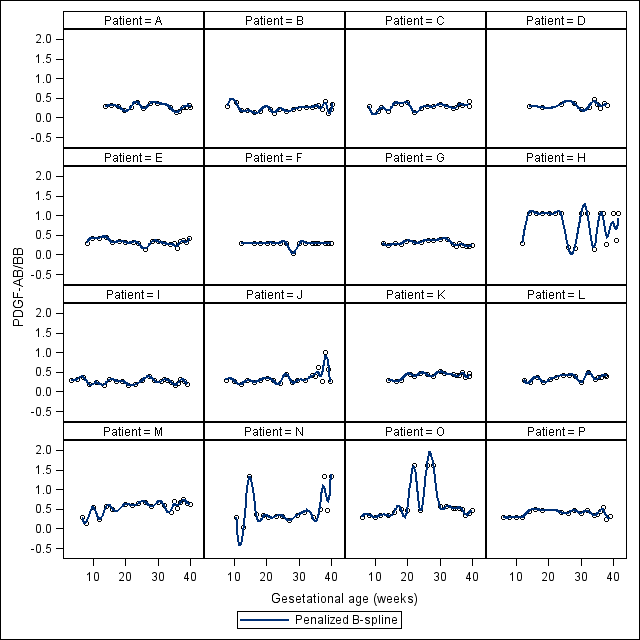


1. RANTES


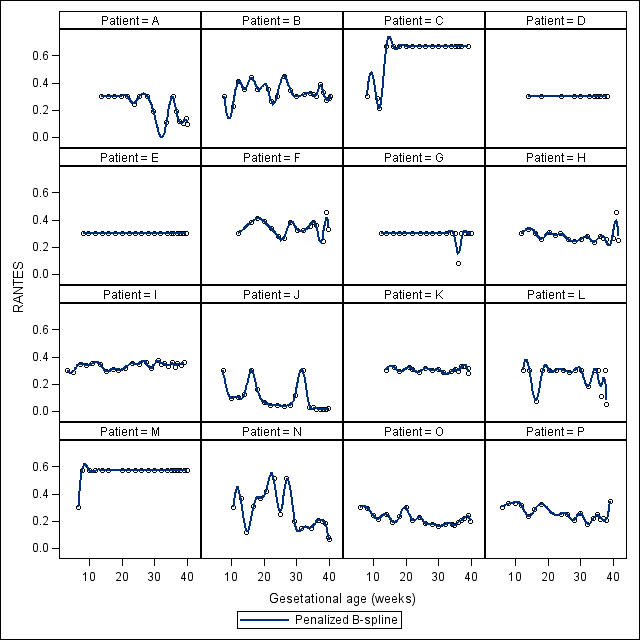


1. sCD40L


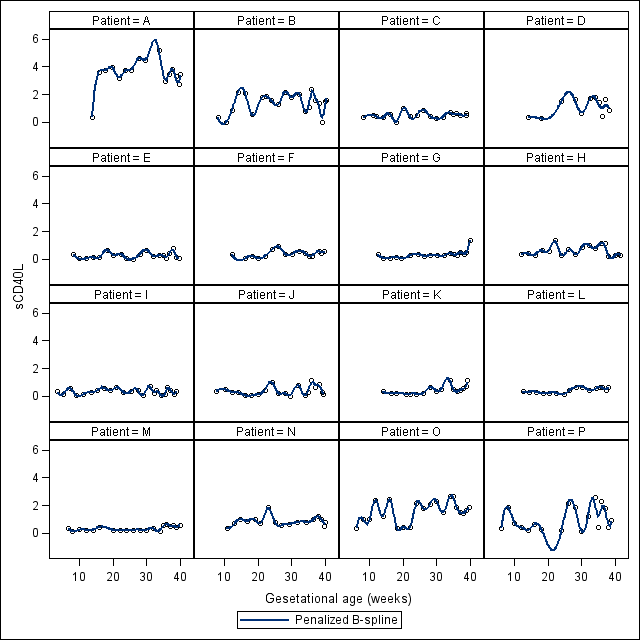


1. s-IL2Rα


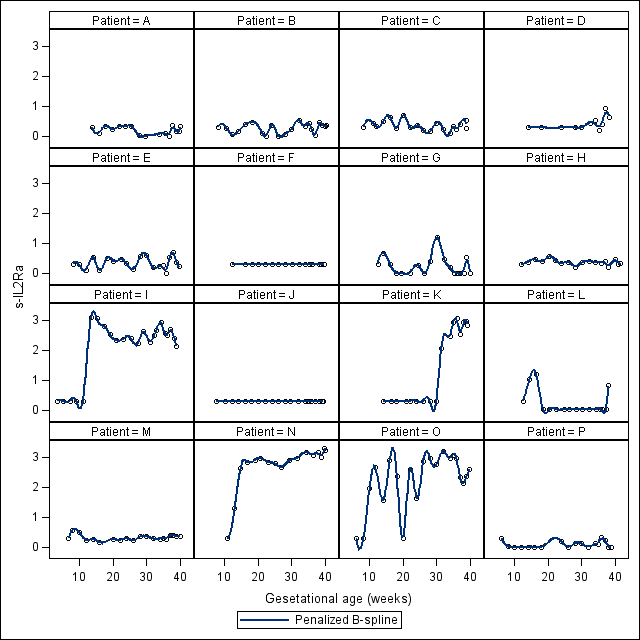


1. TGFα


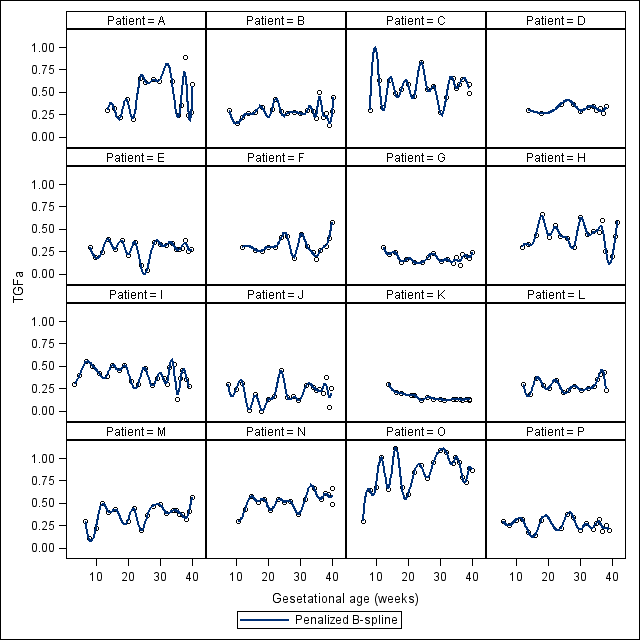


1. TNFα


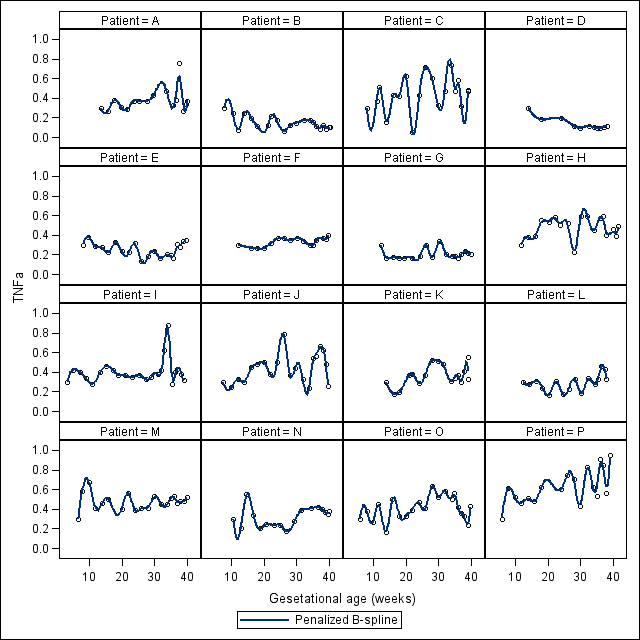


1. TNFβ


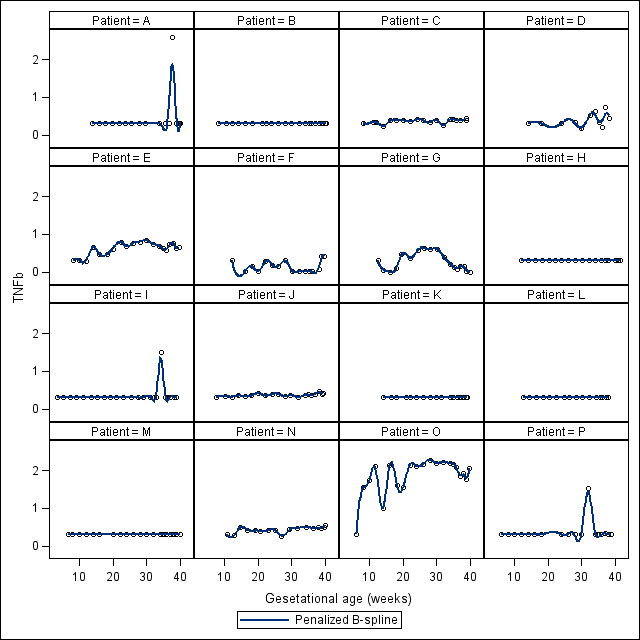


1. VEGF


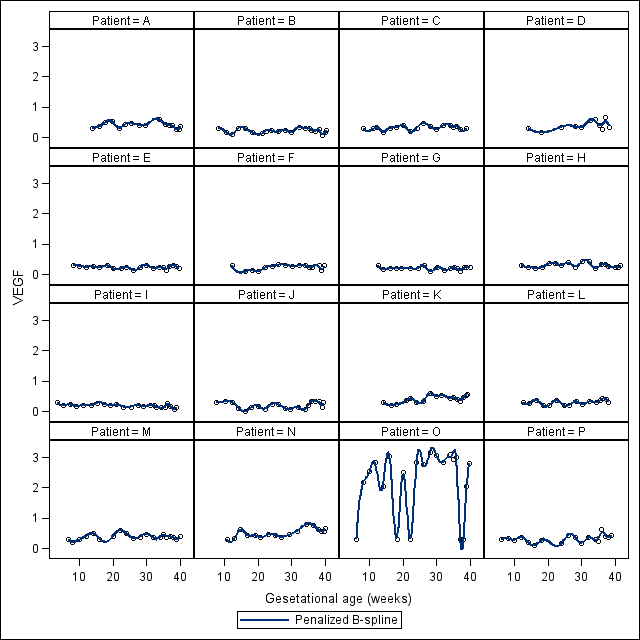


**Figure S2. Superimposed growth curve of MDC.**


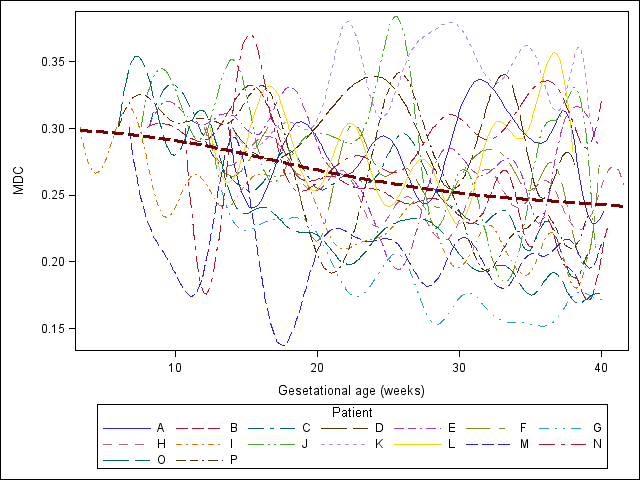


**Figure S3. Supimposed growth curve of IL-1β.**


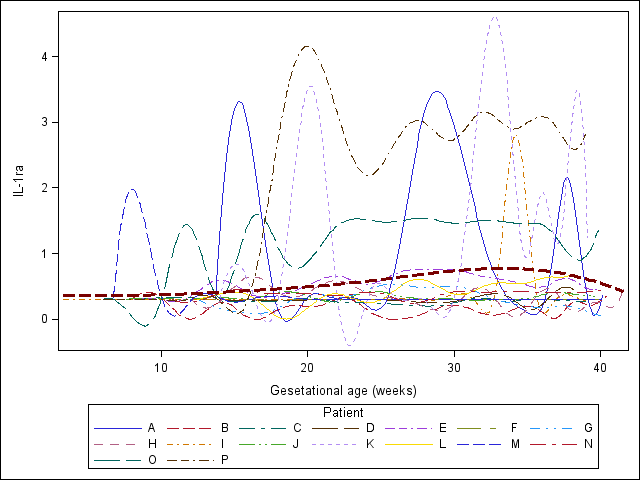

Supplement: Supplementary file 1 — Supplemental material accompanying this article incluldes detailed results of the number of samples with analyte concentrations below or above detection limits, interclass correlation coefficients for each cytokine, nonparametric empiric growth trajectories of individual cytokines with plots separated by individual patients, and the superimposed growth curves of MDC and IL-1b. [file 952571.f1.docx]
